# Enormous diversity of RNA viruses in economic crustaceans

**DOI:** 10.1128/msystems.01016-24

**Published:** 2024-09-27

**Authors:** Xuan Dong, Fanzeng Meng, Chengyan Zhou, Juan Li, Tao Hu, Yiting Wang, Guohao Wang, Jingfei Luo, Xuan Li, Shufang Liu, Jie Huang, Weifeng Shi

**Affiliations:** 1 State Key Laboratory of Mariculture Biobreeding and Sustainable Goods, Yellow Sea Fisheries Research Institute, Chinese Academy of Fishery Sciences; Laboratory for Marine Fisheries Science and Food Production Processes, Qingdao Marine Science and Technology Center; Key Laboratory of Maricultural Organism Disease Control, Ministry of Agriculture and Rural Affairs; Qingdao Key Laboratory of Mariculture Epidemiology and Biosecurity, Qingdao, China; 2 Key Laboratory of Emerging Infectious Diseases in Universities of Shandong, Shandong First Medical University & Shandong Academy of Medical Sciences, Ji'nan, China; 3 Network of Aquaculture Centres in Asia-Pacific, Bangkok, Thailand; 4 Shanghai Institute of Virology, Shanghai Jiao Tong University School of Medicine, Shanghai, China; 5 Ruijin Hospital, Shanghai Jiao Tong University School of Medicine, Shanghai, China; University of Technology Sydney, Glebe, New South Wales, Australia

**Keywords:** crustaceans, virome, diversity, evolution

## Abstract

Crustaceans are important food sources worldwide and possess significant ecological status in the marine ecosystem. However, our understanding of the diversity and evolution of RNA viruses in crustaceans, especially in economic crustaceans, is still limited. Here, 106 batches of economic crustaceans including 13 species were collected from 24 locations in China during 2016–2021. We identified 90 RNA viruses, 69 of which were divergent from the known viruses. Viral transcripts were assigned to 18 different viral families/clades and three unclassified groups. Among the identified viruses, five were double-stranded RNA viruses, 74 were positive-sense single-stranded RNA (+ssRNA) viruses, nine were negative-sense single-stranded RNA (−ssRNA) viruses, and two belonged to an unclassified RNA virus group. Phylogenetic analyses showed that crustacean viruses were often clustered with viruses identified from invertebrates. Remarkably, most crustacean viruses were closely related to those from different host species along the same food chain or ecological aquatic niche. In addition, the genome structures of the newly discovered picornaviruses exhibited remarkable diversity. Our study significantly expands the diversity of viruses in important economic crustaceans and provides essential data for the risk assessment of the pathogens spreading in the global aquaculture industry.

**IMPORTANCE:**

The study delves into the largely uncharted territory of RNA viruses in crustaceans, which are not only vital for global food supply but also play a pivotal role in marine ecosystems. Focusing on economic crustaceans, the research uncovers 90 RNA viruses, with 69 being potentially new to science, highlighting the vast unknown viral diversity within these marine organisms. The findings reveal that these viruses are often related to those found in other invertebrates and tend to share close relationships with viruses from species within the same food web or habitat. This suggests that viruses may move between different marine species more frequently than previously thought. The discovery of such a wide variety of viruses, particularly the diverse genome structures of newly identified picornaviruses, is a significant leap forward in understanding the crustacean virology. This knowledge is crucial for managing disease risks in aquaculture and maintaining the balance of marine ecosystems.

## INTRODUCTION

Crustaceans, named for the protective “armor” that encases their bodies, encompass a diverse range of invertebrates that are globally traded and consumed as food sources (1, 2). These species have adapted to inhabit nearly every conceivable niche within aquatic environments (3). Economically important crustaceans are primarily sourced from aquaculture or wild capture. For instance, species such as *Penaeus vannamei*, *P. chinensis*, *P. monodon*, and *Macrobrachium rosenbergii* are extensively cultivated, yielding substantial economic benefits (4).

Commercial aquatic crustacean farming and fishing have been rising in recent decades, and several countries in Asia and the Americas have a thriving crustacean farming industry (1, 2). However, losses from diseases, especially viral infections, have been increasing in crustacean farming (2, 5). Many important crustacean viruses have been described, including those from families *Bunyaviridae*, *Nimaviridae*, *Nudiviviridae*, *Parvoviridae*, *Picornaviridae*, and unclassified clades (6–23), with more yet to be discovered.

The restricted perspective that viruses are harmful to human and animal health (24–26) has resulted in a skewed picture of viral diversity and their ecological functions. The increasing deployment of meta-transcriptome sequencing has ushered in a revolution in virus discovery. With the discovery of more and more viruses from invertebrates to vertebrates (24, 26–28), terrestrial to aquatic species (9, 10, 29–31), farmed to wild birds (32), and even various game mammals (30), our understanding of the structure, size, and diversity of the virosphere has greatly deepened (24, 25, 33) and we have realized that the known viruses only represent a small subset of the virosphere.

While a few crustaceans inhabit terrestrial environments, the majority live in aquatic settings. They feed on phytoplankton and constitute the base of the food chain. These creatures serve not only as crucial secondary producers in both marine and freshwater ecosystems (34) but also as important virus carriers. Some species, such as *Eriocheir sinensis*, can even migrate along a long distance, potentially facilitating the exchange of viruses between marine and terrestrial environments (35). To date, a plethora of novel RNA viruses have been identified in crustaceans (10, 24), including *Carcinus maenas* (36), European brown shrimp (*Crangon crangon*) (10), signal crayfish (*Pacifastacus leniusculus*) (29), square lobster (*Shinkaia crosnieri*), and some shallow sea soft beetles (9). These findings have enhanced our understanding of virus diversification and distribution patterns in the ocean, and have begun to unveil the hidden diversity of RNA viruses in crustaceans. Importantly, some have been proven to be distinctly pathogenic to their crustacean hosts.

To reveal the unexplored virosphere in economic crustaceans, we collected 106 batches of crustaceans from 13 species in eastern China during 2016–2021, and employed meta-transcriptomic sequencing to (1) uncover the virome composition in economic crustaceans, and to (2) describe the phylogenetic relationships of the newly discovered viruses.

## RESULTS

### Meta-transcriptomic sequencing and identification of the crustaceans

**Fig 1:**
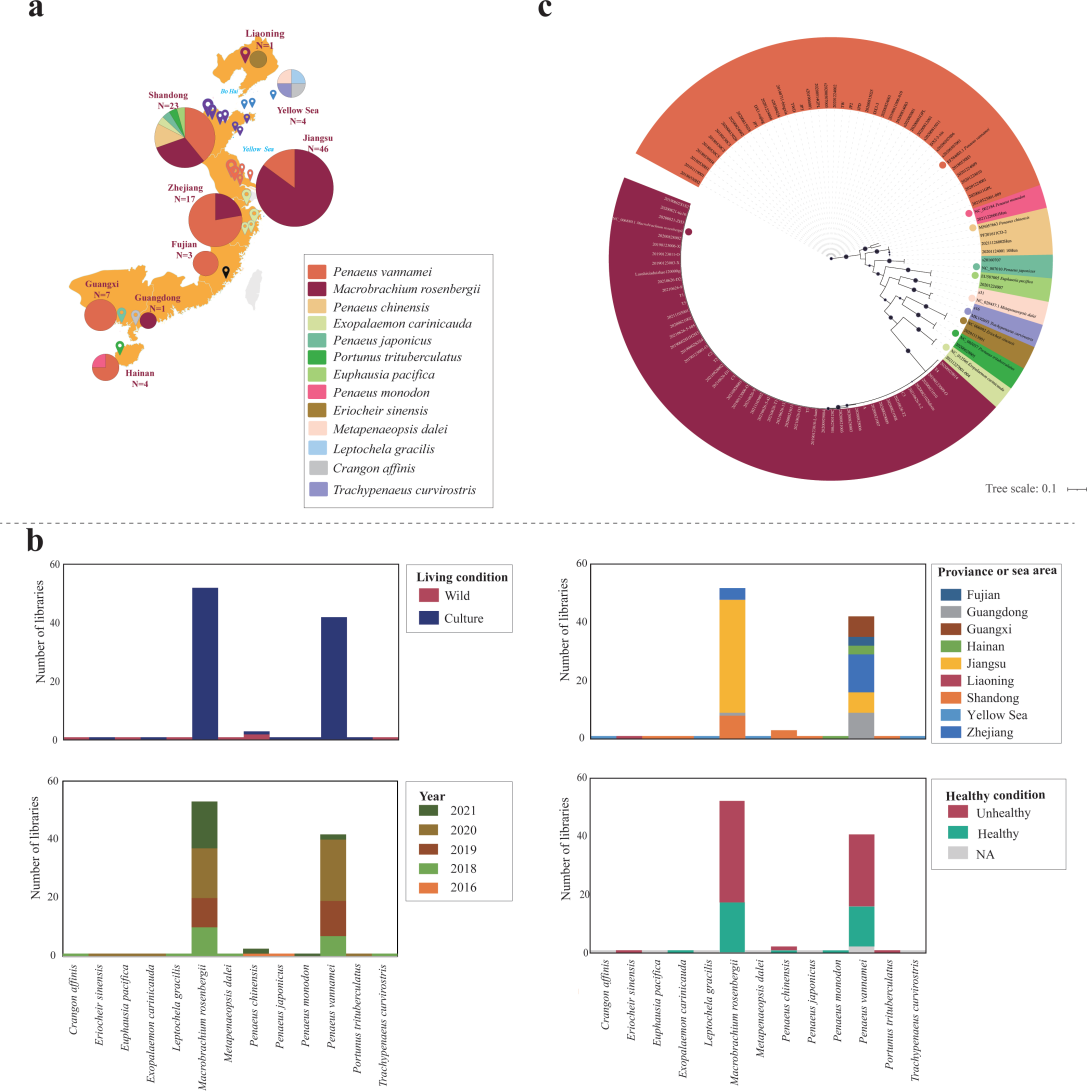
The distribution of crustaceans and the viruses identified in this study. (a) Geographic distribution of the crustaceans surveyed between July 2016 and December 2021 (*N* = 106 libraries). The pie chart showed the number and proportion of the species sampled in the eight provinces and one sea area. Different colors represented different host species. (b) Phylogenetic relationships of the cytochrome C oxidase subunit I (*cox1*) gene sequences of crustacean species in this study. Different colors indicate different host species. (c) Distribution of the samples by living condition, health condition (middle panel), sampling location (bottom panel), and sampling year. See also Table S1.

One hundred and six batches of crustaceans were collected from 24 locations in eight provinces of China and the Yellow Sea between 2016 and 2021, with the top three provinces including Jiangsu (*n* = 46), Shandong (*n* = 23), and Zhejiang (*n* = 17) (Fig. 1a; Table S1). A total of 106 *lnc*RNA libraries were constructed and sequenced. Next-generation sequencing generated a total of 4.00E+09 paired-end reads of raw data, with the number of reads ranging from 1.29E+07 to 7.35E+07 across libraries (Table S1). Among them, there were seven libraries belonging to six wild species. The remaining 99 libraries were from aquaculture species, and Jiangsu had the largest number of libraries (Fig. 1b; Table S1). Of particular note was that 63 libraries consisted of diseased samples, 34 libraries were from healthy individuals, and the health status was not documented for the remaining nine libraries (Fig. 1b; Fig. S1a; Table S1).

The species of the crustaceans were first identified by field experts when sampling, and the cytochrome C oxidase subunit I (*cox1*) gene was subsequently obtained and analyzed to identify the species of the samples (Fig. 1c). A total of 13 species were identified, including *P. vannamei* (*n* = 42), *M. rosenbergii* (*n* = 51), *Euphausia pacifica* (*n* = 1), *P. chinensis* (*n* = 3), *Exopalaemon carinicauda* (*n* = 1), *C. affinis* (*n* = 1), *Portunus trituberculatus* (*n* = 1), *P. japonicus* (*n* = 1), *P. monodon* (*n* = 1), *Leptochela gracilis* (*n* = 1), *Metapenaeopsis dalei* (*n* = 1), *Trachysalambria curvirostris* (*n* = 1), and *E. sinensis* (*n* = 1) (Fig. 1c; Fig. S1; Table S1).

### Composition and diversity of the economic crustacean virome

**Fig 2:**
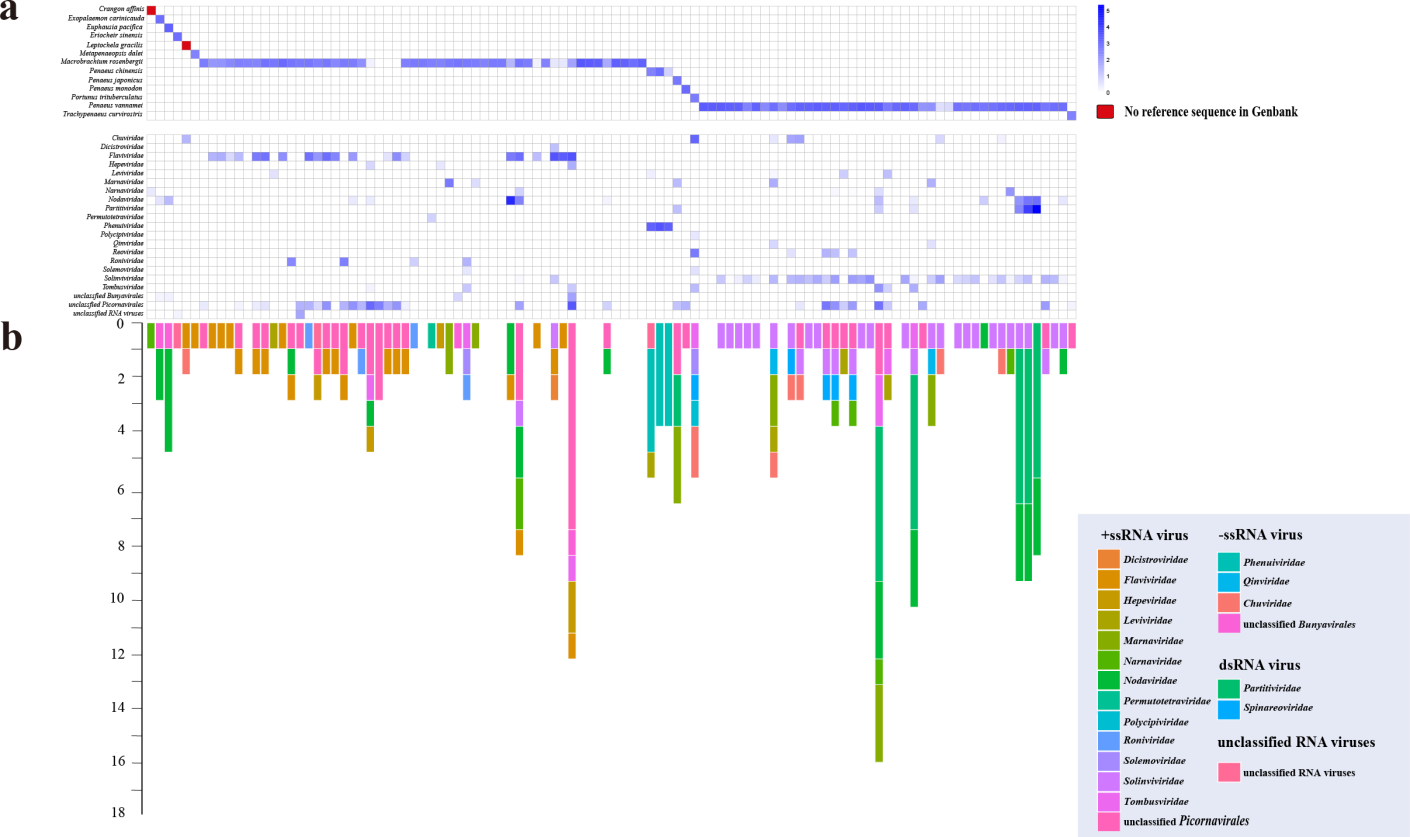
Crustacean viruses were analyzed in this study. (a) Distribution and abundance of crustaceans-associated viruses across different hosts. The relative abundance of the viruses in each library was calculated and normalized by the number of FPKM. Viral species from 18 families, 1 unclassified *Picornavirales*, 1 unclassified *Bunyavirales*, and 1 unclassified RNA virus were shown, with viral taxonomy (family) and host abundance indicated by different colors. “No reference sequence in GenBank” means that the *cox1* reference sequence for the related species has not been made available in the GenBank. (b) Number of virus species in the corresponding libraries (Fig. 2a), with viral taxonomy (family) indicated by different colors.

After *de novo* assembly and mapping, 246 consensus sequences of viral contigs were obtained (Table S2). They were then clustered at 95% average nucleotide identity and over 80% of the sequence length, producing 102 species-level viral populations (viral operational taxonomic units, vOTUs) (37). They included 90 RNA-dependent RNA polymerase (RdRp) vOTUs and 12 vOTUs encoding coat, glycoprotein, or hypothetical protein. All the viral assemblies containing the RdRp domain had ≥4-fold coverage, with the highest reaching 30,711 folds (Table S2). We verified the viruses using reverse transcription-polymerase chain reaction (RT-PCR) and Sanger sequencing (Fig. S1a). All the 90 viruses were RNA viruses with different genome structures: double-stranded (ds) RNA viruses (*n* = 5), negative-sense single-stranded (−ss) RNA viruses (*n* = 9), positive-sense single-stranded (+ss) RNA viruses (*n* = 74) (Fig. 2b; Table 1), and two unclassified viruses. The number of genomic segments of the 90 viruses ranged from one to four, including 82 viruses with unsegmented genomes and eight viruses with segmented genomes (Table S2). These viruses showed 22.97%–100% amino acid (aa) identities to their closest relatives in the RdRp protein (Table 1). Sixty-nine out of the 90 virus sequences were divergent from the known viruses, which shared less than 90% aa identity with known viruses in the RdRp protein (38) (Table 1). The abundance of the viruses substantially varied across libraries, ranging from 7.77E−02 to 4.09E+03 fragments per kilobase of exon model per million mapped fragments (FPKM) (Table S2).

**TABLE 1:** Discovery of novel and known viruses in this study

No.	Virus/isolate/(segment)	vOTU/segment no.	Positive library	Classification	Closest relative	Coverage (segment containing RdRp)	aa identity (RdRp^*b*^)	FPKM
Double-sense RNA viruses								
1	Penaeus japonicus partiti-like virus 1	2	1	*Partitiviridae*	Beihai partiti-like virus 9	296	52.31%	2.06E+01
2	Penaeus vannamei reo-like virus 1	1	4	*Spinareoviridae*	Wenling reo-like virus 1	210	42.11%	2.06E+01
3	Penaeus vannamei partiti-like virus 1	2	5	*Partitiviridae*	Fusarium mangiferae partitivirus 1	1,201	70.29%	1.18E+02
4	Penaeus vannamei partiti-like virus 2^*a*^	4	5	*Partitiviridae*	Brine shrimp partiti-like virus 2	228	100.00%	1.58E+01
5	Portunus trituberculatus reo-like virus 1	1	1	*Spinareoviridae*	Beihai reo-like virus 1	4,939	27.58%	5.87E+02
Negative-sense single-stranded RNA viruses								
1	Euphausia pacifica bunya-like virus 1	1	1	Unclassified *Bunyavirales*	Shahe bunya-like virus 3	18	23.09%	1.91E+00
2	Exopalaemon carinicauda bunya-like virus 1	1	1	Unclassified *Bunyavirales*	Wenling crustacean virus 8	31	22.97%	1.79E+00
3	Leptochela gracilis chuvirus-like virus 1	1	1	*Chuviridae*	Herr Frank virus-1	630	50.00%	3.44E+01
4	Macrobrachium rosenbergii bunya-like virus 1	1	2	Unclassified *Bunyavirales*	Beihai shrimp virus 3	258	24.06%	7.88E+01
5	Penaeus vannamei chuvirus-like virus 1	1	4	*Chuviridae*	Sanxia atyid shrimp virus 4	684	71.61%	7.65E+01
6	Penaeus vannamei chuvirus-like virus 2	1	1	*Chuviridae*	Hubei chuvirus-like virus 1	46	39.94%	3.24E+00
7	Penaeus vannamei qinvirus-like virus 1	1	2	*Qinviridae*	Xinzhou nematode virus 3	28	26.42%	4.25E+00
8	Portunus trituberculatus chuvirus-like virus 1	2	1	*Chuviridae*	Wenzhou crab virus 2	7,896	88.04%	1.10E+02
9	Oriental wenrivirus 1^*a*^	4	3	*Phenuiviridae*	Bunyavirales sp.	7,860	100.00%	1.38E+03
Positive-sense single-stranded RNA viruses								
1	Beihai levi-like virus 30^*a*^	1	**1**	*Leviviridae*	Beihai levi-like virus 30	36	100.00%	1.50E+00
2	Beijing sediment noda-like virus 3^*a*^	2	4	*Nodaviridae*	Beijing sediment noda-like virus 3	29	100.00%	1.80E+00
3	Covert mortality nodavirus^*a*^	2	7	*Nodaviridae*	Covert mortality nodavirus	1,030	100.00%	8.50E+01
4	Crangon affinis narna-like virus 1	1	1	*Narnaviridae*	Hubei narna-like virus 12	36	68.96%	2.77E+00
5	Crangon crangon flavivirus^*a*^	1	2	Flaviviridae	Crangon crangon flavivirus	14	100.00%	4.79E−01
6	Crustacea hepe-like virus 1^*a*^	1	1	*Hepeviridae*	Crustacea hepe-like virus 1	149	100.00%	3.67E+01
*7*	Euphausia pacifica noda-like virus 1	2	1	*Nodaviridae*	Hainan forest noda-like virus	89	49.15%	1.14E+01
8	Infectious precocity virus^*a*^	1	23	*Flaviviridae*	Infectious precocity virus	30,711	100.00%	3.87E+03
9	Penaeus vannamei picorna-like virus 2	1	1	Unclassified *Picornavirales*	Beihai picorna-like virus 101	143	31.25%	1.29E+01
10	Macrobrachium rosenbergii golda virus^*a*^	1	4	Roniviridae	Macrobrachium rosenbergii Golda virus	6,975	100.00%	4.75E+02
11	Macrobrachium rosenbergii hepe-like virus 1	1	1	*Hepeviridae*	Sanya hepevirus 1	92	54.71%	6.60E+00
12	Macrobrachium rosenbergii hepe-like virus 2	1	1	*Hepeviridae*	Eastern mosquitofish hepevirus	60	42.86%	1.18E+01
13	Macrobrachium rosenbergii hepe-like virus 3	1	1	Hepeviridae	Forsythia suspensa hepe-like virus	33	47.00%	3.23E+00
14	Macrobrachium rosenbergii hepe-like virus 4	1	1	*Hepeviridae*	Wenzhou shrimp virus 3	1,857	55.56%	1.09E+00
15	Macrobrachium rosenbergii levi-like virus 1	1	1	*Leviviridae*	ssRNA phage SRR5466364_3	42	75.77%	4.28E+00
16	Portunus trituberculatus picorna-like virus 1	1	1	*Unclassified Polycipiviridae*	Red panda picorna-like virus	49	31.78%	3.17E+00
17	Penaeus japonicus picorna-like virus 2	1	1	Unclassified *Picornavirales*	Picornavirales sp.	44	32.88%	2.51E+00
18	Macrobrachium rosenbergii narna-like virus 1	1	1	*Narnaviridae*	Wuhan Millipede virus 4	34	65.24%	2.18E+00
19	Macrobrachium rosenbergii narna-like virus 2	1	1	*Narnaviridae*	Hubei narna-like virus 1	87	53.29%	6.15E+00
20	Macrobrachium rosenbergii noda-like virus 1	1	1	*Nodaviridae*	Wenzhou noda-like virus 1	30	89.88%	2.95E+00
21	Macrobrachium rosenbergii permutotetra-like virus 1^*a*^	1	1	*Permutotetraviridae*	Sanya permutotetravirus 3	97	91.08%	9.26E+00
22	Macrobrachium rosenbergii picorna-like virus 8	1	1	Unclassified *Picornavirales*	Picornavirales Q_sR_OV_036	29	37.17%	6.63E+00
23	Penaeus vannamei marna-like virus 1	1	1	*Marnaviridae*	Fur seal picorna-like virus	29	40.00%	2.49E+00
24	Macrobrachium rosenbergii picorna-like virus 10	1	1	Unclassified *Picornavirales*	Corey virus	26	41.56%	1.92E+00
25	Penaeus vannamei marna-like virus 2	1	1	*Marnaviridae*	Beihai picorna-like virus 14	50	42.96%	6.11E+00
26	Macrobrachium rosenbergii picorna-like virus 5	1	1	Unclassified *Picornavirales*	Changjiang picorna-like virus 5	53	46.74%	5.60E+00
27	Macrobrachium rosenbergii picorna-like virus 11	1	1	Unclassified *Picornavirales*	Wenzhou channeled applesnail virus 3	226	47.00%	7.80E+01
28	Penaeus vannamei picorna-like virus 4	1	5	Unclassified *Picornavirales*	Beihai picorna-like virus 91	5,527	48.72%	6.74E+02
29	Penaeus vannamei picorna-like virus 5	1	1	Unclassified *Picornavirales*	Beihai mantis shrimp virus 3	5,739	50.00%	9.19E+02
30	Macrobrachium rosenbergii picorna-like virus 1	1	1	Unclassified *Picornavirales*	Beihai sipunculid worm virus 5	26	50.39%	2.11E+00
31	Macrobrachium rosenbergii picorna-like virus 2	1	2	Unclassified *Picornavirales*	Ginkgo biloba picorna-like virus	35	52.88%	2.75E+00
32	Macrobrachium rosenbergii picorna-like virus 3	1	1	Unclassified *Picornavirales*	Picornavirales sp.	28	41.42%	1.60E+00
33	Macrobrachium rosenbergii solemo-like virus 1	1	1	*Solemoviridae*	Barns Ness beadlet anemone sobemo-like virus 1	37	40.96%	3.09E+00
34	Macrobrachium rosenbergii tombus-like virus 1^*a*^	1	1	*Tombusviridae*	Ihi tombusvirus	19	95.98%	1.74E+00
35	Macrobrachium rosenbergii tombus-like virus 2	1	1	*Tombusviridae*	Changjiang tombus-like virus 18	60	45.15%	1.27E+01
36	Macrobrachium rosenbergii tombus-like virus 3	1	1	*Tombusviridae*	Olive mild mosaic virus	189	35.16%	1.40E+01
37	Macrobrachium rosenbergii picorna-like virus 6	1	1	Unclassified *Picornavirales*	Ginkgo biloba picorna-like virus	57	53.47%	1.43E+01
38	Penaeus japonicus picorna-like virus 1	1	1	Unclassified *Picornavirales*	Wuhan spider virus 4	216	54.71%	1.57E+01
39	Penaeus chinensis levi-like virus 1	1	1	*Leviviridae*	Wuhan Millipede virus 4	31	37.16%	2.37E+00
40	Macrobrachium rosenbergii picorna-like virus 4	1	3	*Marnaviridae*	Hainan forest noda-like virus	34	55.06%	2.77E+00
41	Macrobrachium rosenbergii picorna-like virus 9	1	1	Unclassified *Picornavirales*	Wenzhou picorna-like virus 40	46	56.61%	1.23E+01
42	Penaeus vannamei picorna-like virus 6	1	1	*Marnaviridae*	Aurantiochytrium single-stranded RNA virus 01	17	59.85%	1.82E+00
43	Penaeus japonicus marna-like virus 2	1	1	*Marnaviridae*	Beihai picorna-like virus 15	42	60.39%	1.91E+00
44	Penaeus vannamei marna-like virus 3	1	1	*Marnaviridae*	Hubei picorna-like virus 4	45	61.66%	4.46E+00
45	Macrobrachium rosenbergii picorna-like virus 7	1	1	Unclassified *Picornavirales*	Wenzhou picorna-like virus 40	68	63.76%	1.89E+01
46	Penaeus vannamei levi-like virus 1	1	1	*Leviviridae*	ssRNA phage SRR5466725_22	360	60.45%	2.22E+01
47	Penaeus vannamei levi-like virus 2	1	1	*Leviviridae*	Changjiang levi-like virus 3	112	57.51%	1.08E+01
48	Penaeus vannamei picorna-like virus 1	1	1	Unclassified *Picornavirales*	Cragig virus 7	33	68.70%	3.12E+00
49	Penaeus japonicus marna-like virus 1	1	1	*Marnaviridae*	Marine RNA virus BC-4	36	70.00%	1.44E+00
50	Penaeus monodon picorna-like virus 1	1	1	Unclassified *Picornavirales*	Beihai shrimp virus 2	476	70.97%	3.92E+01
51	Macrobrachium rosenbergii dicistro-like virus 1	1	1	*Dicistroviridae*	Hubei picorna-like virus 24	197	71.61%	2.00E+01
52	Penaeus japonicus marna-like virus 3	1	1	*Marnaviridae*	Beihai picorna-like virus 39	12	72.78%	6.58E−01
53	Penaeus vannamei narna-like virus 1	1	1	*Narnaviridae*	Beihai narna-like virus 10	1,839	45.31%	1.54E+02
54	Penaeus vannamei narna-like virus 2	1	1	Narnaviridae	Aspergillus flavus narnavirus 1	16	31.02%	1.25E+00
55	Penaeus vannamei narna-like virus 3	1	1	*Narnaviridae*	Beihai narna-like virus 6	4	76.63%	5.38E+00
56	Penaeus vannamei narna-like virus 4	1	1	*Narnaviridae*	Changjiang narna-like virus 2	15	28.41%	1.50E+00
57	Penaeus vannamei noda-like virus 1	1	2	*Nodaviridae*	Beihai noda-like virus 5	114	58.69%	1.14E+01
58	Penaeus vannamei noda-like virus 2^*a*^	1	5	*Nodaviridae*	Brine shrimp noda virus 3	83	98.34%	8.84E+00
59	Penaeus vannamei picorna-like virus 3	1	1	Unclassified *Picornavirales*	Beihai picorna-like virus 102	631	73.12%	6.53E+01
60	Penaeus vannamei marna-like virus 5	1	2	*Marnaviridae*	Beihai picorna-like virus 9	52	76.64%	2.95E+00
61	Penaeus vannamei marna-like virus 4	1	2	*Marnaviridae*	Wenzhou picorna-like virus 50	809	81.70%	5.07E + 01
62	Macrobrachium rosenbergii marna-like virus 2	1	1	*Marnaviridae*	Hubei picorna-like virus 4	78	89.17%	8.45E+00
63	Trachypenaeus curvirostris picorna-like virus 1^*a*^	1	1	Unclassified *Picornavirales*	Dicistroviridae sp.	36	93.93%	1.73E+00
64	Wenzhou shrimp virus 8^*a*^	1	31	*Solinviviridae*	Wenzhou shrimp virus 8	747	100.00%	1.74E+01
65	Penaeus vannamei tombus-like virus 1	1	3	*Tombusviridae*	Caledonia beadlet anemone tombus-like virus 1	96	68.86%	9.16E+00
66	Penaeus vannamei tombus-like virus 2	1	1	*Tombusviridae*	Kummerowia striata luteovirus	13	23.76%	1.29E+00
67	Macrobrachium rosenbergii marna-like virus 1^a^	1	1	*Marnaviridae*	Kummerowia striata marnavirus	71	98.39%	7.22E+00
68	Picornaviridae sp.^*a*^	1	1	Unclassified *Picornavirales*	Picornaviridae sp.	183	98.50%	5.19E+01
69	Portunus trituberculatus solemo-like virus 1	1	1	*Solemoviridae*	Beihai sobemo-like virus 15	43	45.19%	4.45E+00
70	Portunus trituberculatus tombus-like virus 1	1	1	*Tombusviridae*	Wenzhou crab virus 4	340	73.58%	2.70E+01
71	Macrobrachium rosenbergii virus 1^*a*^	1	2	*Solinviviridae*	Macrobrachium rosenbergii virus 1	150	99.00%	1.46E+01
72	Macrobrachium rosenbergii virus 15^*a*^	1	2	Unclassified *Picornavirales*	Macrobrachium rosenbergii virus 15	206	99.71%	6.23E+01
73	Wenzhou picorna-like virus 41^*a*^	1	1	Unclassified *Picornavirales*	Wenzhou picorna-like virus 41	216	100.00%	2.46E+01
74	Wenzhou channeled applesnail virus 2^*a*^	1	15	Unclassified *Picornavirales*	Wenzhou channeled applesnail virus 2	12,389	100.00%	1.37E+03
NA								
1	Eriocheir sinensis virus 1	1	1	Unclassified RNA viruses	Beihai blue swimmer crab virus 1	32	53.47%	1.70E+00
2	Shahe isopoda virus 5^*a*^	1	1	Unclassified RNA viruses	Shahe isopoda virus 5	675	100.00%	6.98E+01

^*a*^ Known virus.

^*b*^ RdRp, RNA-dependent RNA polymerase; FPKM, fragments per kilobase of exon model per million mapped fragments. The largest one of aa identity, coverage, and abundance is shown if detected in multiple libraries.

In detail, the 90 RNA viruses could be classified into 18 different viral families and three unclassified groups, including *Chuviridae* (*n* = 4), *Dicistroviridae* (*n* = 1)*, Flaviviridae* (*n* = 2), *Hepeviridae* (*n* = 5), *Leviviridae* (*n* = 5), *Marnaviridae* (*n* = 12)*, Narnaviridae* (*n* = 7), *Nodaviridae* (*n* = 6), *Partitiviridae* (*n* = 3), *Permutotetraviridae* (*n* = 1), *Phenuiviridae* (*n* = 1)*, Polycipiviridae* (*n* = 1), *Qinviridae* (*n* = 1), *Roniviridae* (*n* = 1), *Solemoviridae* (*n* = 2), *Solinviviridae* (*n* = 2), *Spinareoviridae* (*n* = 2), *Tombusviridae* (*n* = 6), unclassified *Bunyavirales* (*n* = 3)*,* unclassified *Picornavirales* (*n* = 23), and unclassified RNA viruses (*n* = 2) (Fig. 2a; Fig. S1a; Table 1).

### Phylogenetic analysis of the RNA viruses identified in economic crustaceans: double-stranded RNA viruses

**Fig 3:**
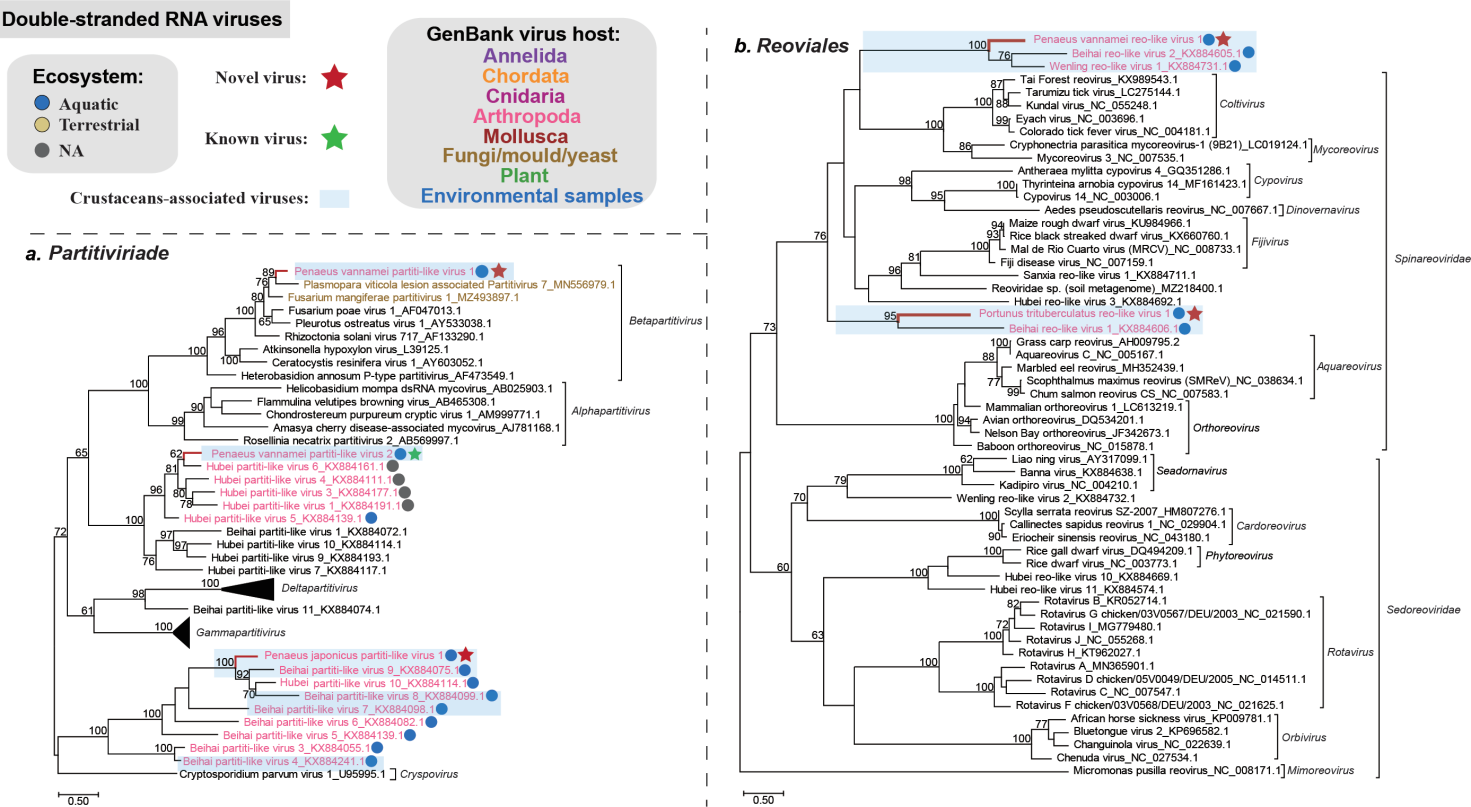
Maximum likelihood phylogenetic analyses of the RdRp protein sequences of the double-sense RNA viruses identified in crustaceans. Multiple sequence alignment was performed using MAFFT v7.407 with the E-INS-I algorithm. Non-conserved amino acid regions were removed using Trimal v.1.4. We selected the best-fit amino acid substitution model identified using ModelFinder and performed phylogenetic analyses using the maximum likelihood method embedded in IQ-TREE v.2.1.4 with 1,000 bootstrap replicates. Red bold lines indicate the viruses found in this study. Taxon names of the reference sequences downloaded from GenBank within the same cluster with crustacean viruses found in this study were colored by the apparent host group from which the viral sequence originated. Additionally, the reference crustacean viruses within the same cluster as those found in this study were highlighted. The host ecosystem was distinguished by different colored solid circles. All the phylogenetic trees were mid-point rooted for clarity and only bootstrap values ≥60% were shown. The viruses identified in this study were highlighted in red and bold. Best-fit amino acid substitution model: (a) Q.pfam + F + R6; (b) Q.pfam + F + R4.

Our data showed the presence of five dsRNA viruses from two families. Among them, three viruses (Penaeus vannamei partiti-like virus 1, Penaeus japonicus partiti-like virus 1, and Penaeus vannamei partiti-like virus 2) with segmented genomes fell within the family *Partitiviridae* (Fig. 3a). Penaeus vannamei partiti-like virus 1 fell with the genus *Betapartitivirus*. Penaeus japonicus partiti-like virus 1 and Penaeus vannamei partiti-like virus 2 fell within two unclassified lineages of *Partitiviridae* and clustered with representative viruses identified from various arthropods within the phylum Arthropoda (Fig. 3a) living in aquatic and terrestrial environments. The RdRp protein sequences of Penaeus vannamei partiti-like virus 1, Penaeus japonicus partiti-like virus 1, and Penaeus vannamei partiti-like virus 2 showed 70.29%, 52.31%, and 100% aa identity to their most related strains in the RdRp protein, respectively (Table 1).

Phylogenetic analysis revealed that two viruses (Penaeus vannamei reo-like virus 1 and Portunus trituberculatus reo-like virus 1) fell within the family *Spinareoviridae* (39), order *Reovirales* with a segmented genome (Fig. 3b). Penaeus vannamei reo-like virus 1 and Portunus trituberculatus reo-like virus 1 clustered into two independent branches with other crustacean viruses, respectively, which did not fall within the currently defined genera of the family *Spinareoviridae*.

### Negative-sense RNA viruses

**Fig 4:**
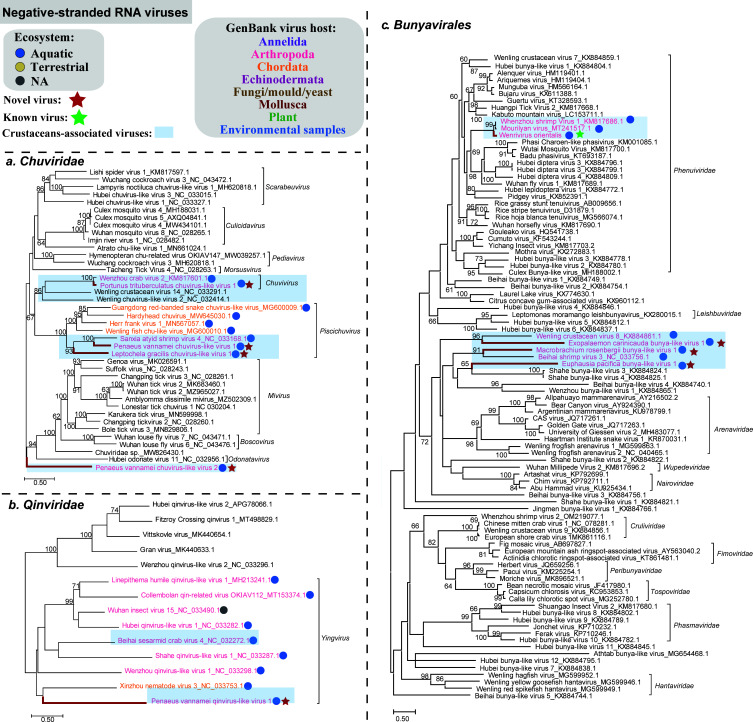
Maximum likelihood phylogenetic analyses of the RdRp protein sequences of the negative-sense RNA viruses identified in crustaceans. Multiple sequence alignment was performed using MAFFT v.7.407 with the E-INS-I algorithm. Non-conserved amino acid regions were removed using Trimal v.1.4. We selected the best-fit amino acid substitution model identified using ModelFinder and performed phylogenetic analyses using the maximum likelihood method embedded in IQ-TREE v.2.1.4 with 1,000 bootstrap replicates. Red bold lines indicate the viruses found in this study. Taxon names of the reference sequences downloaded from GenBank within the same cluster with crustacean viruses found in this study were colored by the apparent host group from which the viral sequence originated. Additionally, the reference crustacean viruses within the same cluster as those found in this study were highlighted. The host ecosystem was distinguished by different colored solid circles. All the phylogenetic trees were mid-point rooted for clarity and only bootstrap values ≥60% were shown. The viruses identified in this study were highlighted in red and bold. Best-fit amino acid substitution model: (a) LG + I + G4; (b) LG + F + I + G4; (c) Q.pfam + F + R7.

Nine −ssRNA viruses identified here fell into four groups: *Chuviridae*, *Qinviridae*, *Phenuiviridae*, and unclassified *Bunyavirales* (Fig. 4). Four −ssRNA viruses belonged to the family *Chuviridae*, falling within three different lineages (Fig. 4a). Penaeus vannamei chuvirus-like virus 1 and Leptochela gracilis chuvirus-like virus 1 belonged to *Piscichuvirus*. Penaeus vannamei chuvirus-like virus 2 might represent an unclassified chuvirus. Portunus trituberculatus chuvirus-like virus 1 had two genomic segments, which were grouped with Wenzhou crab virus 2 of the genus *Chuvivirus*, sharing aa identity of 88.04% for RdRp (Table S2). One −ssRNA virus, Penaeus vannamei qinvirus-like virus 1, belonged to the genus *Yingvirus*, family *Qinviridae,* and clustered together with Xinzhou nematode virus 3 but with low bootstrap support, and they shared an aa identity of 26.42% in RdRp (Fig. 4b).

Four bunyaviruses were identified in this study. Among them, oriental wenrivirus 1, identified in *P. chinensis*, belonged to the family *Phenuiviridae*. Exopalaemon carinicauda bunya-like virus 1, Macrobrachium rosenbergii bunya-like virus 1, and Euphausia pacifica bunya-like virus 1 were unclassified crustacean bunyaviruses, forming a crustacean virus cluster together with several previously described bunya-like viruses (Fig. 4c).

### Positive-sense RNA viruses

Seventy-four +ssRNA viruses from 13 families and one unclassified *Picornavirales* were identified, including one *Dicistroviridae* virus, two *Flaviviridae* viruses, five *Hepeviridae* viruses, five *Leviviridae* viruses, 12 *Marnaviridae* viruses, seven *Narnaviridae* viruses, six *Nodaviridae* viruses, one *Permutotetraviridae* virus, one *Polycipiviridae* virus, one *Roniviridae* virus, two *Solinviviridae v*iruses, two *Solemoviridae* viruses, six *Tombusviridae* viruses, and 23 unclassified *Picornavirales* viruses (Table 1).

**Fig 5:**
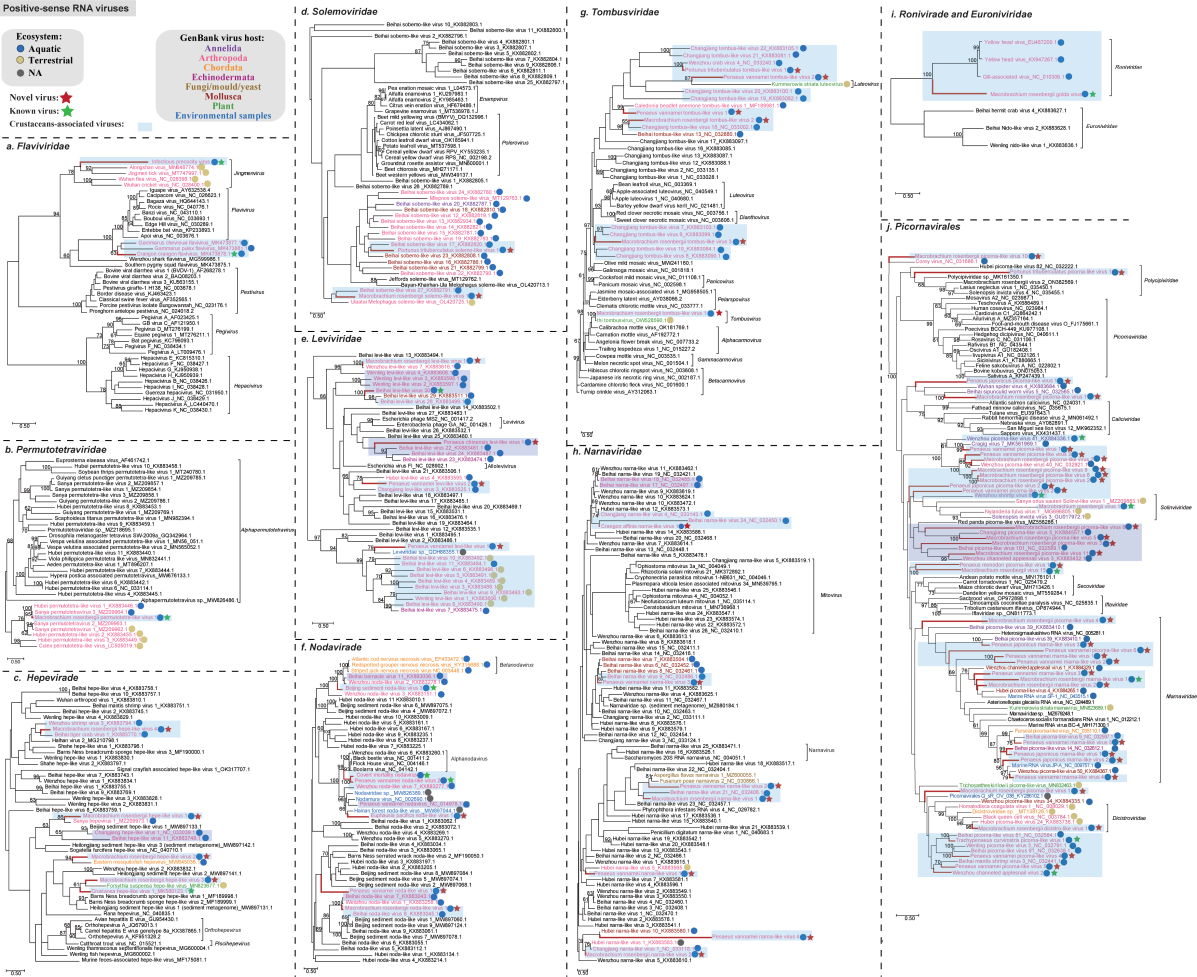
Maximum likelihood phylogenetic analyses of the RdRp protein sequences of the positive-sense RNA viruses identified in crustaceans. Multiple sequence alignment was performed using MAFFT v.7.407 with the E-INS-I algorithm. Non-conserved amino acid regions were removed using Trimal v.1.4. We selected the best-fit amino acid substitution model identified using ModelFinder and performed phylogenetic analyses using the maximum likelihood method embedded in IQ-TREE v.2.1.4 with 1,000 bootstrap replicates. Red bold lines indicate the viruses found in this study. Taxon names of the reference sequences downloaded from GenBank within the same cluster with crustacean viruses found in this study were colored by the apparent host group from which the viral sequence originated. Additionally, the reference crustacean viruses within the same cluster as those found in this study were highlighted. The host ecosystem was distinguished by different colored solid circles. All the phylogenetic trees were mid-point rooted for clarity and only bootstrap values ≥60% were shown. Viruses identified in this study were highlighted in red and bold. Best-fit amino acid substitution model: (a) Q.pfam + F + R5, (b) LG + G4, (c) LG + I + G4, (d) Q.pfam + G4, (e) LG + F + I + G4, (f) Q.pfam + F + I + G4, (g) LG + I + G4, (h) PMB + R5, (i) LG + F + I + G4, (j) Q.pfam + F + R6.

Two known flaviviruses, infectious precocity virus (IPV) and Crangon crangon flavivirus (CcFV), were identified in this study. Consistent with previous studies, IPV was identified from diseased *M. rosenbergii*, clustered together with jingmenviruses (Fig. 5a). Crangon crangon flavivirus, a virus infecting *C. crangon*, was detected from *L. gracilis* (40)*,* which had the closest relationship with some flaviviruses from aquatic hosts (Fig. 5a). The RdRp protein identity between Macrobrachium rosenbergii permutotetra-like virus 1, a permutotetravirus identified from *M. rosenbergii,* and Sanya permutotetravirus 3 was 91.08% (Table 1). Phylogenetic analysis showed that they clustered together with several arthropod permutotetraviruses and belonged to an unclassified group within *Permutotetraviridae* (Fig. 5b).

As expected, most viruses from the water environment, whether they were invertebrate or vertebrate viruses, were grouped together. We found a virus related to vertebrate virus in this analysis. Macrobrachium rosenbergii hepe-like virus 2, discovered in *M. rosenbergii,* belonged to the family *Hepeviridae* and clustered together with Eastern mosquitofish hepevirus discovered in *Gambusia holbrooki* (Fig. 5c), with an aa identity of 42.86% in RdRp (Table 1). Viruses from the family *Solemoviridae* are important pathogens of major crops (41), and two solemoviruses were identified in this study. Portunus trituberculatus solemo-like virus 1 fell within an aquatic virus lineage including viruses from aquatic arthropods, mollusks, and worms (Fig. 5d). In addition, five leviviruses were identified in this study. Apart from Penaeus vannamei levi-like virus 1, the other four viruses clustered with leviviruses from arthropod hosts (Fig. 5e).

Two known nodaviruses, covert mortality nodavirus (CMNV) and Penaeus vannamei noda-like virus 2 (PvNV2) were identified in this study, which clustered together. One nodavirus, Euphausia pacifica noda-like virus 1 (EpNV1), was grouped with the Hainan forest noda-like virus, which was found from soil in a forest in Hainan, China (Fig. 5f). Notably, the aa identity between PvNV2 and brine shrimp noda virus 3, a noda-like virus identified from brine shrimp, was 98.34% in RdRp. EpNV1 showed 49.15% aa identity with previously identified Hainan forest noda-like virus in RdRp. Regarding the remaining two noda-like viruses, Penaeus vannamei noda-like virus 1 and Macrobrachium rosenbergii noda-like virus 1, which belonged to uncharacterized noda-like viruses together with Beijing sediment noda-like virus 3, they exhibited aa identities of 58.69% and 89.88% with their closest counterparts in RdRp, respectively (Fig. 5f; Table 1).

Furthermore, six tombus-like viruses have been identified in this study, including Macrobrachium rosenbergii tombus-like virus variants 1, 2, and 3, Penaeus vannamei tombus-like virus variants 1 and 2, and Portunus trituberculatus tombus-like virus 1. Except for Macrobrachium rosenbergii tombus-like virus 1, sharing 95.98% aa identity in RdRp with Ihi tombusvirus which primarily infects terrestrial plants, the other five tombusviruses displayed identities ranging from 23.76% to 73.58% with their closest counterparts in RdRp. Notably, Penaeus vannamei tombus-like virus 2 was also found in a cluster associated with viruses infecting terrestrial plants (Fig. 5g).

A total of seven narna-like viruses were discovered, including Penaeus vannamei narna-like virus 1–4, Macrobrachium rosenbergii narna-like virus 1 and 2, and Crangon affinis narna-like virus 1. Notably, Penaeus vannamei narna-like virus 2 and 4 were divergent from previously documented viruses, and only showed 31.02% and 28.41% aa identities with their closest relatives in RdRp, respectively. The remaining five viruses shared identities ranging from 45.31% to 76.63% with their closest relatives in RdRp (Table 1). Phylogenetic analyses revealed that Penaeus vannamei narna-like virus 1 and 2 clustered together with terrestrial arthropod viruses (Hubei narna-like virus 5) and fungi viruses (Aspergillus flavus narnavirus 1 and Fusarium poae narnavirus 2), respectively (Fig. 5h). Penaeus vannamei narna-like virus 3 clustered with several mollusk viruses and Beihai narna-like virus 9, with the latter infecting horseshoe crabs. Macrobrachium rosenbergii narna-like virus 1 and 2, as well as Crangon affinis narna-like virus 1, clustered with previously identified marine arthropod viruses (Fig. 5h). However, Penaeus vannamei narna-like virus 4 formed a cluster with Hubei narna-like virus 10, which is associated with *Unio douglasiae* infection (Fig. 5h). In addition, we identified a known virus belonging to the family *Roniviridae*, Macrobrachium rosenbergii golda virus (Fig. 5i), from four libraries (Fig. S1).

A total of 39 picornaviruses were characterized in this study from six crustacean species. These picornaviruses were categorized into several distinct clades in the order *Picornavirales* according to phylogenetic analysis of the RdRp sequences, including *Marnaviridae*, *Dicistroviridae*, *Polycipiviridae*, *Solinviviridae*, and unclassified *Picornavirales* (Fig. 5j). Thirty-one newly identified picornaviruses showed 31.25% to 89.17% aa identities with their closest relatives in RdRp (Table 1). Wenzhou shrimp virus 8 (WzSV8) and Macrobrachium rosenbergii virus 1 clustered with various viruses that infect terrestrial arthropods within the family *Solinviviridae* (Fig. 5j). Similarly, Macrobrachium rosenbergii dicistro-like virus 1 fell within the family *Dicistroviridae* and also clustered with several viruses infecting terrestrial arthropods (Fig. 5j). Notably, Macrobrachium rosenbergii picorna-like virus 1 and Penaeus japonicus picorna-like virus 1 formed a separate branch with viruses infecting two marine annelids. Furthermore, Macrobrachium rosenbergii picorna-like virus 2, 6, 7, and 9 and Penaeus vannamei picorna-like virus 3 clustered together, but they did not fall within any established families. The remaining viruses were grouped together with those discovered in Chordata, Cnidaria, Mollusca, Nematoda, fungi, plants, and environmental samples (Fig. 5j).

### Genome structure of the economic crustacean viruses

**Fig 6:**
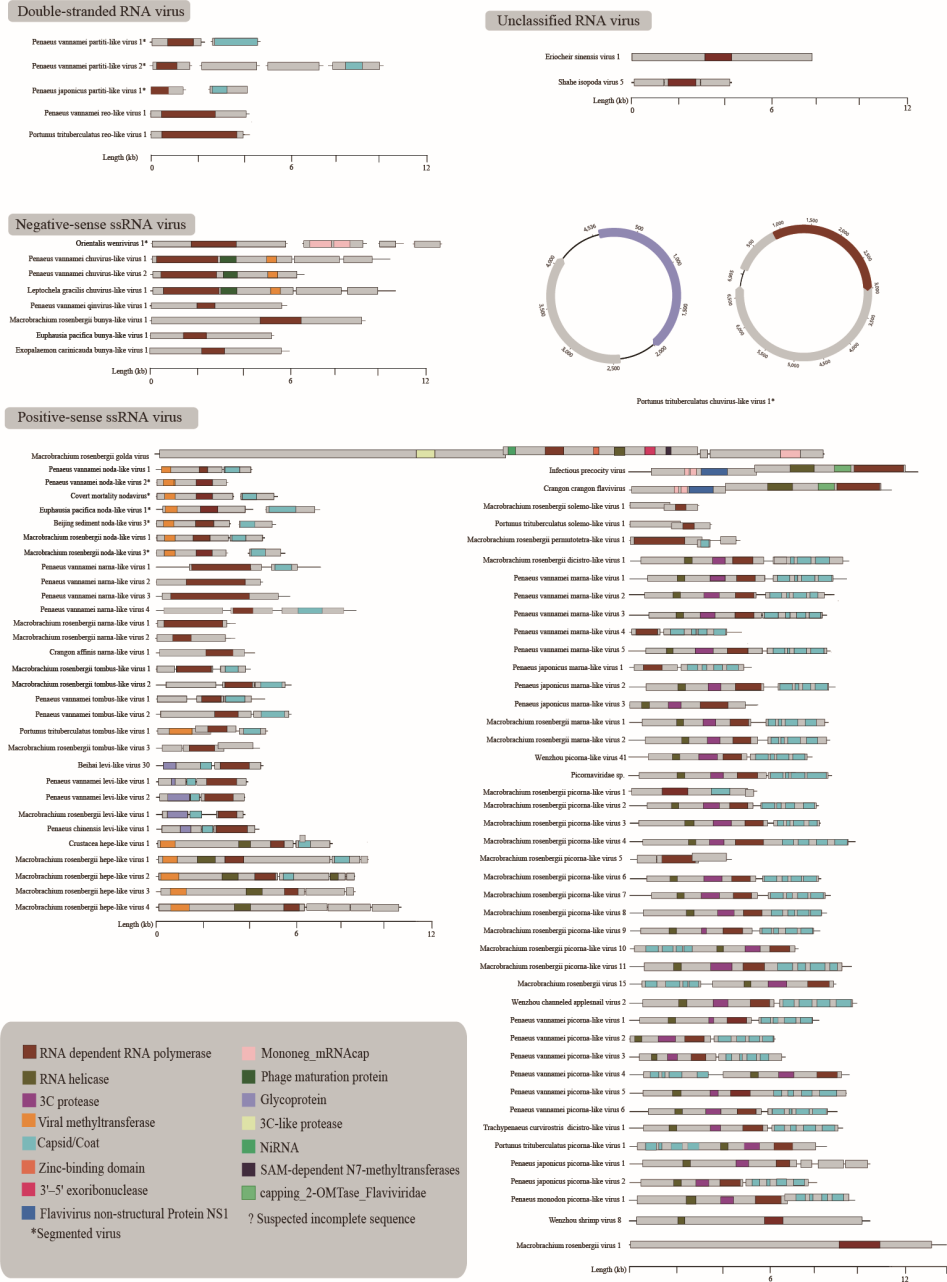
Predicted genome structure of the crustacean viruses. The conserved domains of the viruses were visualized and viruses with segmented genomes were indicated by asterisks. The genomes are depicted at a consistent length scale, as indicated at the bottom of the illustration. Within each genome, the outlined boxes represent the predicted ORF boundaries. Colored boxes highlight the regions that exhibit relatively high BLAST homology to viral proteins/domains, with specifics detailed in the lower left corner of the figure.

Overall, the genome structures of the newly found viruses were typical of their respective viral families/clades (Fig. 6). However, some viruses showed variations in terms of genome length and structures. For example, genomic structures of unclassified *Picornavirales* were different from those of the known members of *Picornavirales*, manifested by the diversity of sequence length, the number of open reading frames (ORFs), and the location of conserved domains (42). Meanwhile, the position of the conserved domains of some picornaviruses was reversed, such as Macrobrachium rosenbergii picorna-like virus 10, Macrobrachium rosenbergii virus 15, Portunus trituberculatus picorna-like virus 1, and Penaeus vannamei picorna-like virus 4 (Fig. 6). Moreover, the ORFs of Penaeus monodon picorna-like virus 1, Macrobrachium rosenbergii picorna-like virus 1, and Macrobrachium rosenbergii picorna-like virus 5 were overlapped.

### Multihost detection of some viruses in this study

Herein, we discovered two nodaviruses that might be able to infect different hosts. CMNV, a nodavirus that may cause deaths in farmed shrimps (13), has been detected from *P. vannamei* (Fig. S1a). However, we found CMNV from wild *E. pacifica* in this study. Another nodavirus, PvNV2, discovered in *P. vannamei*, shared 98.34% aa identity in the RdRp protein with brine shrimp noda virus 3 discovered in brine shrimp (*Artemia*).

CcFV was first reported to infect wild marine *C. crangon* from Weser Estuary, Germany, in 2019 (40). In this study, we detected CcFV in wild *L. gracilis* and *M. dalei* collected in the Yellow Sea of China in November 2018. The CcFV identified from Germany and China shared a nucleotide identity of >99.00%.

Horseshoe crabs are ancient marine and brackish water arthropods of the family Limulidae. Beihai levi-like virus 30 (BhLV30) was a levi-like virus of *Leviviridae*, which was first found in horseshoe crab (24). In this study, BhLV30 was also detected in desalinated *P. vannamei*, and BhLV30 from different hosts shared a nucleotide sequence identity of 99.84%.

In this study, we detected Wenzhou channeled applesnail virus 2 in 15 *M*. *rosenbergii* samples collected from Jiangsu Province. This virus was initially discovered in channeled applesnail in 2016 (24), and Wenzhou channeled applesnail virus 2 from different hosts shared a nucleotide sequence identity of >99.70%. It is noteworthy that both channeled applesnail and *M. rosenbergii* live in the same aquatic habitats.

## DISCUSSION

Our comprehensive survey revealed a high diversity of RNA viruses in economically important crustaceans, including 90 viruses (69 of which were divergent from the known viruses) from 13 crustacean species. Viral transcripts were categorized into 18 distinct viral families or clades, as well as three unclassified RNA viruses. Among them, five were dsRNA viruses, 74 were +ssRNA viruses, nine were −ssRNA viruses, and two were assigned to an unclassified group of RNA viruses.

Notably, the detection of a virus in multiple hosts, as observed in PvNV2 and CcFV, could have important implications for the stability and health of aquatic ecosystems. The presence of mollusk-associated viruses in crustaceans suggests a potential transmission route through the food chain. This is further supported by the detection of Wenzhou channeled applesnail virus 2 in both channeled applesnail and *M. rosenbergii*, which share the same aquatic habitat. Some viruses were grouped together with those discovered in Chordata, Cnidaria, Mollusca, Nematoda, fungi, plants, and environmental samples such as viruses from the order *Picornavirales* (Fig. 5j). This suggests that these viruses might have originated from the crustaceans’ diet or an interconnected broader food chain. These findings highlight the complexity of the aquatic ecosystem and the potential for direct/indirect transmission of viruses through the food chain or the environment. However, the potential ecological implications of these viruses such as their impact on multihost detection and biological invasions (43, 44) warrant further investigation. It should be noted that environmental contamination, surface adhesion, and processing contamination might also lead to the detection of a virus in multiple hosts.

This study might also have important implications for disease management in the crustacean industry. Current strategies for disease prevention and control primarily focus on known pathogens. However, the discovery of viruses in economic crustaceans suggests that these strategies may not be sufficient, and approaches considering viral diversity and potential viral transmission are favorable. In particular, the discovery of CMNV in *E. pacifica* highlights the potential biosecurity risk associated with the use of live feed in aquaculture. The findings underscore the importance of biosecurity in aquaculture practices. Measures such as quarantine of live feed, regular health checks, and rapid response to disease outbreaks may help to prevent and mitigate the spread of viral infections.

In sum, our systematic investigation provided valuable insights into the diversity of RNA viruses in economic crustaceans. These findings underscore the need for improved biosecurity measures and viral surveillance in aquaculture farms to prevent disease outbreaks and ensure the sustainability of the crustacean industry. However, the existence of a highly diverse virome highlights the challenges in elucidating the evolution, etiology, and ecology of viruses in crustaceans and poses a serious threat to the ecological breeding of healthy crustaceans.

## MATERIALS AND METHODS

### Sample collection and species identification

In total, 106 batches of economic crustaceans were collected between 2016 and 2021 from 24 different sampling sites covering eight provinces and one sea area (Fig. 1a; Table S1). In order to determine the species, crustacean species were first identified by experienced aquatic biologists. Then, *cox1* sequences downloaded from GenBank based on aquatic biologists’ judgment were utilized as reference sequences for mapping. Subsequently, multiple sequence alignment was performed between the sequenced *cox1* sequences and the *cox1* reference sequences using MAFFT v.7.407 with the E-INS-I algorithm (45, 46). Non-conserved amino acid regions were then removed using Trimal v.1.4 (47). We selected the best-fit amino acid substitution model identified using ModelFinder (48) and performed phylogenetic analyses using the maximum likelihood method embedded in IQ-TREE v.2.1.4 with 1,000 bootstrap replicates (49). Finally, phylogenetic analysis of the *cox1* gene was conducted to further confirm the species of the samples. Some crustaceans showed signs of diseases such as empty stomach and midgut and sexual precocity (Table S1). All samples were stored at the Yellow Sea Fisheries Research Institute, Chinese Academy of Fishery Sciences, Qingdao, China.

### RNA library construction and meta-transcriptomic sequencing

Total RNA extraction, removal of ribosomal RNA, construction of RNA libraries, and meta-transcriptomic sequencing were performed as previously described (12). Briefly, liquid nitrogen was used to grind the crustacean samples, and then the Trizol reagent was used to extract total RNA (Invitrogen, Carlsbad, CA, USA). Ninety-nine libraries were sequenced with a lncRNA sequencing strategy (Table S1). Ribosomal RNA was removed using the Epicentre Ribo-zero rRNA Removal Kit (Epicentre, Madison, WI, USA) and then lncRNA libraries were prepared using the NEB Next Ultra RNA Library Prep Kit for Illumina (NEB, USA). The other seven libraries were sequenced using a mRNA sequencing strategy (Table S1), with the NEB Next Ultra RNA Library Prep Kit for Illumina (NEB, USA). 150 bp paired-end read sequencing of the RNA libraries was performed on the NovaSeq 6000 (Illumina, USA) or HiseqX sequencing platforms (Table S1). The library construction and sequencing were performed by Novogene (Beijing, China).

### Sequence assembly and RNA virus discovery

For each library, the raw sequencing reads were adaptor- and quality-trimmed using the Fastp v.0.20.0 (50, 51) program. Clean reads were *de novo* assembled directly using Trinity v.2.5.1 with default parameter settings (52). To obtain potential virus contigs and to remove false positives, all the assembled contigs were compared against the non-redundant protein database (nr) downloaded on 22 February 2022 from GenBank by BLASTn (53) and BLASTx (54), with a cutoff E-value of 1 × 10^−5^, and the organisms were limited to viruses to remove potential host, plant, bacterial, and fungal sequences (24, 26). For the hits, we excluded false-positive contigs that did not contain any ORF. The remaining hits were thought to be potential virus contigs and were then merged to form longer viral contigs using Bowtie2-2.3.5.1-Linux-x86_64 (55) in “local” model and “end to end” model. We then used Geneious v.2021.2.2 (56) (https://www.geneious.com) to display the mapping results. The mapped virus consensus sequences were used to obtain the species-level vOTU using cd-hit 4.8.1 (57, 58). To confirm the assembly results of the full-length genomes, reads were mapped onto them using low sensitivity and faster parameters in Geneious Primer, and the number of reads mapped to each target genome was assessed and presented in the column “Virus Reads” in Table S2.

### Viral genome confirmation and annotation

To verify the virus sequences, we performed RT-PCR with primers designed according to the assembled viral contigs (Table S3). Primer-BLAST (59, 60) (https://www.ncbi.nlm.nih.gov/tools/primer-blast/) was used to design the PCR primers. Reverse transcription was performed at 30°C for 10 min, 42°C for 60 min, and 70°C for 15 min with the random 6-mers and oligo dT using the PrimeScript II 1^ST^ Strand cDNA Synthesis Kit (TaKaRa). PCR was then performed in a 25 µL mixture containing 12.5 µL *EX Taq* mix (TaKaRa) (with 0.625 U *EX Taq*, 5 nmol deoxynucleotide triphosphate (dNTP), and 37.5 nmol MgCl_2_), 10 pmol forward/reverse primers, and 1 µL cDNA template. PCR was initiated at 95°C for 5 min, followed by 40 cycles at 95°C for 30 s, 55°C for 50 s, and 72°C for 40 s, ending at 72°C for 7 min. The amplicons were analyzed and sequenced after 1% agarose gel electrophoresis. Sanger sequencing was performed by Tsingke (Qingdao, China). We prepared one to two sets of specific primers for detecting in order to study potential cross-species transmission events (Table S3). The viruses identified in this study were temporarily named using the common name of host, such as *Penaeus vannamei*, *Macrobrachium rosenbergii*, etc., followed by the classification and number if there is more than one virus in that category. The final virus species names will be determined in consultation with the International Committee on Taxonomy of Viruses.

Except the special start codon of known viruses, putative viral ORFs were predicted by Geneious (v.2021.1.1), and ORFfinder (59, 61) (https://www.ncbi.nlm.nih.gov/orffinder) was used to predict putative ORFs of crustacean viruses with the minimal length of 100 aa, which were further compared with reference sequences, with built-in parameters (genetic code: standard; start codons: ATG). Conserved protein motifs in the sequences were identified by Conserved Domain Search (59, 62) (https://www.ncbi.nlm.nih.gov/Structure/cdd/wrpsb.cgi) and Hhpred (63).

### Phylogenetic analysis of economic crustacean viruses

To infer the evolutionary history of the viruses identified in this study, we used the conserved RdRp protein sequences of the RNA viruses to perform phylogenetic analysis. The closest relatives and representative viral reference sequences of associated genera were retrieved by BLASTx by comparing the viral contigs against the NCBI database. The related virus reference sequences were downloaded from GenBank. Multiple sequence alignment was performed using MAFFT v.7.407 with the E-INS-I algorithm (45, 46). Non-conserved amino acid regions were removed using Trimal v.1.4 (47) with parameter “-gt 1.” We selected the best-fit amino acid substitution model identified using ModelFinder (48) and performed phylogenetic analyses using the maximum likelihood method embedded in IQ-TREE v.2.1.4 with models chosen by the Bayesian information criterion and 1,000 bootstrap replicates (49).

### Abundance analysis of the economic crustacean viruses

In order to investigate the diversity and distribution of the viruses in crustacean samples, the abundance (number of reads) aligned to each of the 246 viral genomes was obtained by mapping the clean reads of each sample to the viral consensus sequences using Bowtie2-2.3.5.1-Linux-x86_64. We then used Samtools v.1.9 (64) and Geneious Primers to summarize and visualize the results. Table S2 displayed the number of sequencing reads mapped to each of the 246 genomes and the mapped read numbers were normalized according to the adjusted FPKM as follows:


$$FPKM=\frac{genome\;reads}{total\;reads\left(millions\right)\times genome\;length\left(kb\right)}$$

## Data Availability

The raw data from the meta-transcriptomic sequencing generated in this study are available at the NCBI Sequence Read Archive (SRA) database under the BioProject accession PRJNA675895 and PRJNA860710. All viral sequences generated in this study have been deposited in GenBank under accession numbers PP054165–PP054195, PP214942–PP214953, PP215143–PP215340, PP210859, PP210860, PP239332, PP239333, PP334143 (Table S2).

## SUPPLEMENTAL MATERIAL

Fig. S1 msystems.01016-24-s0001.pdf The scatter diagram and number of libraries with viruses in crustaceans.

Table S1 msystems.01016-24-s0002.pdf Host and geographic information of the samples.

Table S2 msystems.01016-24-s0003.pdf Diversity of the viruses in crustaceans.

Table S3 msystems.01016-24-s0004.pdf Primers used in this study.

ASM does not own the copyrights to Supplemental Material that may be linked to, or accessed through, an article. The authors have granted ASM a non-exclusive, world-wide license to publish the Supplemental Material files. Please contact the corresponding author directly for reuse.
