# Supplementary material for: Enormous diversity of RNA viruses in economic crustaceans
Source: mSystems. 2024 Sep 27;9(10):e01016-24. doi: 10.1128/msystems.01016-24 (PMC11494968; doi:10.1128/msystems.01016-24)
Supplement: Fig. S1 — The scatter diagram and number of libraries with viruses in crustaceans. [file msystems.01016-24-s0001.pdf]

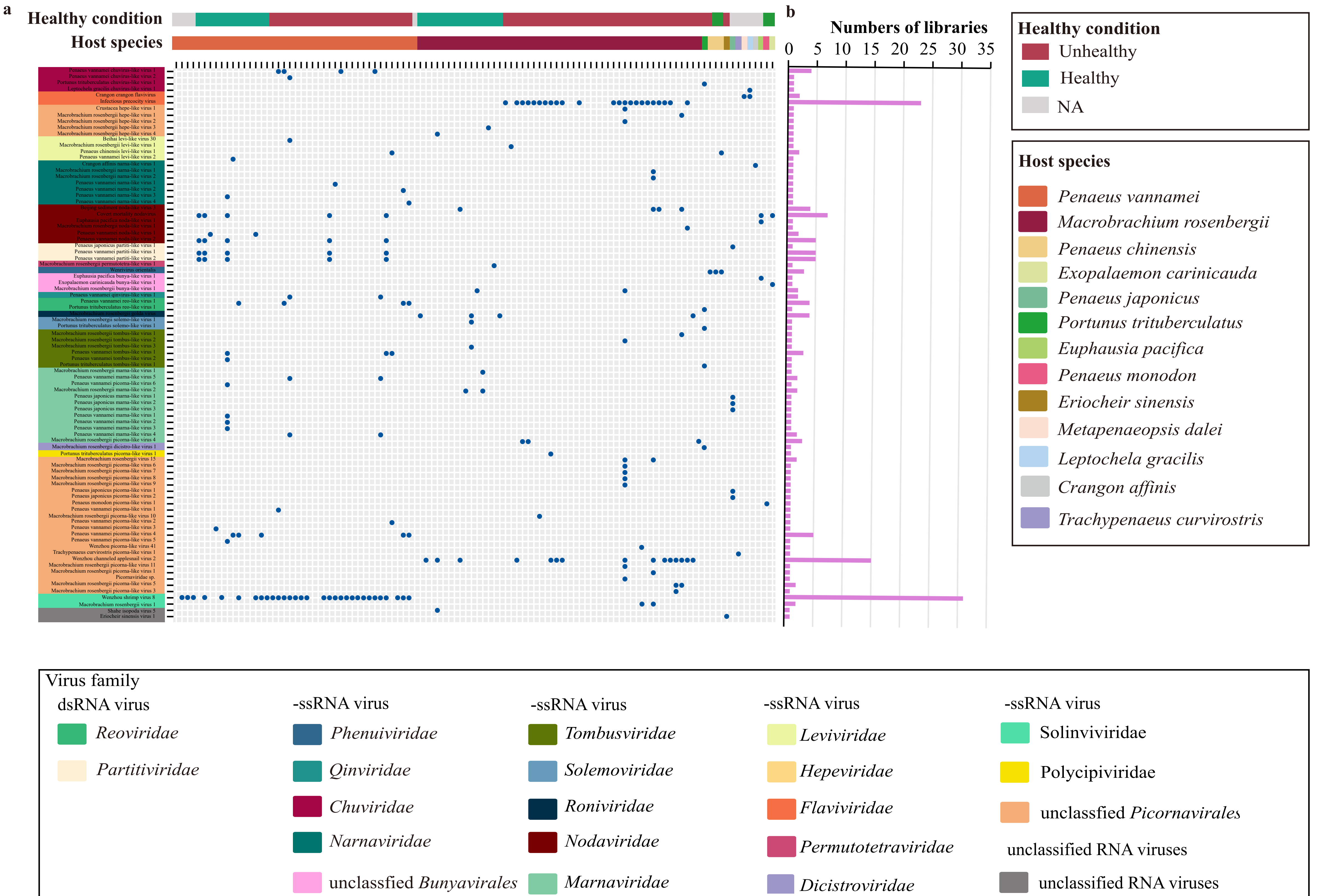
a
Healthy condition
Host species
b
Numbers of libraries
0
5
10
15
20
25
30
35
Healthy condition
Unhealthy
Healthy
NA
Host species
Penaeus vannamei
Macrobrachium rosenbergii
Penaeus chinensis
Exopalaemon carinicauda
Penaeus japonicus
Portunus trituberculatus
Euphausia pacifica
Penaeus monodon
Eriocheir sinensis
Metapenaeopsis dalei
Leptochela gracilis
Crangon affinis
Trachypenaeus curvirostris
Virus family
dsRNA virus
Reoviridae
Partitiviridae
-ssRNA virus
Phenuiviridae
Qinviridae
Chuviridae
Narnaviridae
unclassfied Bunyavirales
-ssRNA virus
Tombusviridae
Solemoviridae
Roniviridae
Nodaviridae
Marnaviridae
-ssRNA virus
Leviviridae
Hepeviridae
Flaviviridae
Permutotetraviridae
Dicistroviridae
-ssRNA virus
Solinviviridae
Polycipiviridae
unclassfied Picornavirales
unclassified RNA viruses
unclassified RNA viruses
