# Supplementary material for: Enormous diversity of RNA viruses in economic crustaceans
Source: mSystems. 2024 Sep 27;9(10):e01016-24. doi: 10.1128/msystems.01016-24 (PMC11494968; doi:10.1128/msystems.01016-24)
Supplement: Table S1 — Host and geographic information of the samples. [file msystems.01016-24-s0002.pdf]

Supplementary Table 1. Host and geographic information of samples

Bioproject accession	Library ID	SRR accession	Sequencing method	Host species	Rawdata	Collection date	Collection location	Healthy condition	Condition	Note	Sample numbers
PRJNA860710	12	SRR27709306	lncRNA\HiseqX	*Crangon affinis*	4.24E+07	201811	Yellow Sea	NA	NA	Collected by epidemiological investigation	1
PRJNA860710	20191119001	SRR27709304	lncRNA\HiseqX	*Penaeus vannamei*	4.46E+07	201911	Hainan Province	NA	NA	Collected by epidemiological investigation	5
PRJNA860710	20200628001	SRR27709291	lncRNA\Novaseq 6000	*Portunus trituberculatus*	3.24E+07	202006	Weifang City, Shandong Provinc	Unhealthy	Sexual precocity	Collected by epidemiological investigation	1
PRJNA860710	20200812001	SRR27709279	lncRNA\Novaseq 6000	*Penaeus vannamei*	2.92E+07	202008	Fujian Province	Unhealthy	Empty stomach; empty intestine	Collected by epidemiological investigation	5
PRJNA860710	20200814003	SRR27709261	lncRNA\Novaseq 6000	*Penaeus vannamei*	3.48E+07	202008	Rudong City, Jiangsu Province	Healthy	NA	Collected by epidemiological investigation	5
PRJNA860710	20200815024	SRR27709249	lncRNA\Novaseq 6000	*Penaeus vannamei*	3.05E+07	202008	Rudong City, Jiangsu Province	Unhealthy	Empty stomach; empty intestine	Collected by epidemiological investigation	5
PRJNA860710	20200815025	SRR27709237	lncRNA\Novaseq 6000	*Penaeus vannamei*	3.64E+07	202008	Rudong City, Jiangsu Province	Healthy	NA	Collected by epidemiological investigation	5
PRJNA860710	20200815026	SRR27709227	lncRNA\Novaseq 6000	*Penaeus vannamei*	3.42E+07	202008	Rudong City, Jiangsu Province	Unhealthy	Empty stomach; empty intestine	Collected by epidemiological investigation	5
PRJNA860710	20200821002	SRR27709218	lncRNA\Novaseq 6000	*Macrobrachium rosenbergii*	2.77E+07	202008	Gaoyou City, Jiangsu Province	Healthy	NA	Collected by epidemiological investigation	1
PRJNA860710	20200821003	SRR27709268	lncRNA\Novaseq 6000	*Macrobrachium rosenbergii*	4.11E+07	202008	Gaoyou City, Jiangsu Province	Healthy	NA	Collected by epidemiological investigation	1
PRJNA860710	20200821007	SRR27709303	lncRNA\Novaseq 6000	*Macrobrachium rosenbergii*	2.48E+07	202008	Gaoyou City, Jiangsu Province	Unhealthy	Sexual precocity	Collected by epidemiological investigation	1
PRJNA860710	20200821008	SRR27709301	lncRNA\Novaseq 6000	*Macrobrachium rosenbergii*	3.58E+07	202008	Gaoyou City, Jiangsu Province	Unhealthy	Sexual precocity	Collected by epidemiological investigation	1
PRJNA860710	20200821010	SRR27709300	lncRNA\Novaseq 6000	*Macrobrachium rosenbergii*	3.53E+07	202008	Gaoyou City, Jiangsu Province	Unhealthy	Sexual precocity	Collected by epidemiological investigation	1
PRJNA860710	20200821011	SRR27709298	lncRNA\Novaseq 6000	*Macrobrachium rosenbergii*	3.45E+07	202008	Gaoyou City, Jiangsu Province	Unhealthy	Sexual precocity	Collected by epidemiological investigation	1
PRJNA860710	20200824001	SRR27709297	lncRNA\Novaseq 6000	*Penaeus vannamei*	2.70E+07	202008	Rudong City, Jiangsu Province	NA	NA	Collected by epidemiological investigation	5
PRJNA860710	20200824002	SRR27709296	lncRNA\Novaseq 6000	*Penaeus vannamei*	2.66E+07	202008	Rudong City, Jiangsu Province	NA	NA	Collected by epidemiological investigation	5
PRJNA860710	20200828002	SRR27709295	lncRNA\Novaseq 6000	*Macrobrachium rosenbergii*	3.77E+07	202008	Gaoyou City, Jiangsu Province	Unhealthy	Sexual precocity	Collected by epidemiological investigation; virus purified solution of tissue m	NA
PRJNA860710	20200828003	SRR27709294	lncRNA\Novaseq 6000	*Macrobrachium rosenbergii*	4.25E+07	202008	Gaoyou City, Jiangsu Province	Unhealthy	Sexual precocity	Collected by epidemiological investigation; virus purified solution of tissue m	NA
PRJNA860710	20200828005	SRR27709293	lncRNA\Novaseq 6000	*Macrobrachium rosenbergii*	3.80E+07	202008	Gaoyou City, Jiangsu Province	Unhealthy	Sexual precocity	Collected by epidemiological investigation; virus purified solution of tissue m	NA
PRJNA860710	20200828006	SRR27709292	lncRNA\Novaseq 6000	*Macrobrachium rosenbergii*	2.98E+07	202008	Gaoyou City, Jiangsu Province	Unhealthy	Sexual precocity	Collected by epidemiological investigation; virus purified solution of tissue m	NA
PRJNA860710	20200929004	SRR27709290	lncRNA\Novaseq 6000	*Macrobrachium rosenbergii*	3.41E+07	202009	Gaoyou City, Jiangsu Province	Unhealthy	Sexual precocity	Collected by epidemiological investigation	1
PRJNA860710	20200929009	SRR27709289	lncRNA\Novaseq 6000	*Macrobrachium rosenbergii*	3.64E+07	202009	Gaoyou City, Jiangsu Province	Healthy	NA	Collected by epidemiological investigation	1
PRJNA860710	20200929014	SRR27709288	lncRNA\Novaseq 6000	*Macrobrachium rosenbergii*	3.29E+07	202009	Gaoyou City, Jiangsu Province	Healthy	NA	Collected by epidemiological investigation	1
PRJNA860710	20201113001	SRR27709287	lncRNA\Novaseq 6000	*Eriocheir sinensis*	3.59E+07	202011	Panjin City, Liaoning Province	Unhealthy	Sexual precocity	Collected by epidemiological investigation	1
PRJNA860710	20201224001	SRR27709285	lncRNA\Novaseq 6000	*Penaeus vannamei*	2.98E+07	202012	Weifang City, Shandong Provinc	Healthy	NA	Collected by epidemiological investigation	5
PRJNA860710	20201224002	SRR27709284	lncRNA\Novaseq 6000	*Penaeus vannamei*	4.06E+07	202012	Weifang City, Shandong Provinc	Unhealthy	Mass mortality	Collected by epidemiological investigation	1
PRJNA860710	20201224006	SRR27709283	lncRNA\Novaseq 6000	*Penaeus vannamei*	3.29E+07	202012	Weifang City, Shandong Provinc	Unhealthy	Mass mortality	Collected by epidemiological investigation	1
PRJNA860710	20201224007	SRR27709282	lncRNA\Novaseq 6000	*Euphausia pacifica*	2.92E+07	202012	Weifang City, Shandong Provinc	NA	NA	Collected by epidemiological investigation	5
PRJNA860710	20201224009	SRR27709281	lncRNA\Novaseq 6000	*Penaeus vannamei*	3.90E+07	202012	Weifang City, Shandong Provinc	Healthy	NA	Collected by epidemiological investigation	5
PRJNA860710	20201224010	SRR27709280	lncRNA\Novaseq 6000	*Penaeus vannamei*	3.47E+07	202012	Weifang City, Shandong Provinc	Unhealthy	growth retardation	Collected by epidemiological investigation	1
PRJNA860710	20210827001	SRR27709278	lncRNA\Novaseq 6000	*Macrobrachium rosenbergii*	3.35E+07	202108	Zhanjiang City, Guangdong Prov	Unhealthy	Sexual precocity	Collected by epidemiological investigation	1
PRJNA860710	20210828001	SRR27709277	lncRNA\Novaseq 6000	*Macrobrachium rosenbergii*	3.60E+07	202108	Huzhou City, Zhejiang Province	Unhealthy	Sexual precocity	Collected by epidemiological investigation	1
PRJNA860710	20210828002	SRR27709276	lncRNA\Novaseq 6000	*Macrobrachium rosenbergii*	3.46E+07	202108	Huzhou City, Zhejiang Province	Unhealthy	Sexual precocity	Collected by epidemiological investigation	1
PRJNA860710	20211103001	SRR27709275	lncRNA\Novaseq 6000	*Macrobrachium rosenbergii*	3.68E+07	202108	Huzhou City, Zhejiang Province	Unhealthy	Sexual precocity	Collected by epidemiological investigation; virus purified solution of tissue m	1
PRJNA860710	20211103002	SRR27709274	lncRNA\Novaseq 6000	*Macrobrachium rosenbergii*	3.86E+07	202108	Huzhou City, Zhejiang Province	Unhealthy	Sexual precocity	Collected by epidemiological investigation; virus purified solution of tissue m	1
PRJNA860710	20180530C1	SRR27709273	mRNA\HiseqX	*Penaeus vannamei*	4.54E+07	201805	Beihai City, Guangxi Province	Healthy	NA	Collected by epidemiological investigation	1
PRJNA860710	20180530C2	SRR27709272	mRNA\HiseqX	*Penaeus vannamei*	4.75E+07	201805	Beihai City, Guangxi Province	Healthy	NA	Collected by epidemiological investigation	1
PRJNA860710	20180530C3	SRR27709271	mRNA\HiseqX	*Penaeus vannamei*	4.88E+07	201805	Beihai City, Guangxi Province	Healthy	NA	Collected by epidemiological investigation	1
PRJNA860710	20180530H1	SRR27709302	mRNA\HiseqX	*Penaeus vannamei*	4.99E+07	201805	Beihai City, Guangxi Province	Unhealthy	Empty stomach; empty intestine	Collected by epidemiological investigation	1
PRJNA860710	20180530H2	SRR27709262	mRNA\HiseqX	*Penaeus vannamei*	5.27E+07	201805	Beihai City, Guangxi Province	Unhealthy	Empty stomach; empty intestine	Collected by epidemiological investigation	1
PRJNA860710	20180530H3	SRR27709305	mRNA\HiseqX	*Penaeus vannamei*	4.67E+07	201805	Beihai City, Guangxi Province	Unhealthy	Empty stomach; empty intestine	Collected by epidemiological investigation	1
PRJNA860710	20180530H4	SRR27709260	mRNA\HiseqX	*Penaeus vannamei*	4.83E+07	201805	Beihai City, Guangxi Province	Unhealthy	Empty stomach; empty intestine	Collected by epidemiological investigation	1
PRJNA675895	20190123003-O	SRR13077183	lncRNA\HiseqX	*Macrobrachium rosenbergii*	4.21E+07	201901	Qingdao City, Shandong Provinc	Healthy	NA	Collected from infection experiment	1
PRJNA860710	20190123003-X	SRR27709258	lncRNA\HiseqX	*Macrobrachium rosenbergii*	4.96E+07	201901	Qingdao City, Shandong Provinc	Healthy	NA	Collected from infection experiment	1
PRJNA675895	20190123006-O	SRR13077188	lncRNA\HiseqX	*Macrobrachium rosenbergii*	4.04E+07	201901	Qingdao City, Shandong Provinc	Unhealthy	Sexual precocity	Collected from infection experiment	1
PRJNA675895	20190123006-X	SRR13077187	lncRNA\HiseqX	*Macrobrachium rosenbergii*	4.73E+07	201901	Qingdao City, Shandong Provinc	Unhealthy	Sexual precocity	Collected from infection experiment	1
PRJNA675895	20190123009-O	SRR13077186	lncRNA\HiseqX	*Macrobrachium rosenbergii*	4.17E+07	201901	Qingdao City, Shandong Provinc	Unhealthy	Sexual precocity	Collected from infection experiment	1
PRJNA860710	20190123010-L-luan	SRR27709257	lncRNA\HiseqX	*Macrobrachium rosenbergii*	4.64E+07	201901	Qingdao City, Shandong Provinc	Unhealthy	Sexual precocity	Collected from infection experiment	NA
PRJNA675895	20190123011-O	SRR13077185	lncRNA\HiseqX	*Macrobrachium rosenbergii*	3.64E+07	201901	Qingdao City, Shandong Provinc	Unhealthy	Sexual precocity	Collected from infection experiment	1
PRJNA675895	20190602H1H2H3	SRR13077192	lncRNA\HiseqX	*Macrobrachium rosenbergii*	4.12E+07	201906	Gaoyou City, Jiangsu Province	Unhealthy	Sexual precocity	Collected by epidemiological investigation	1
PRJNA675895	20190602S1S2	SRR13077191	lncRNA\HiseqX	*Macrobrachium rosenbergii*	3.04E+07	201906	Gaoyou City, Jiangsu Province	Unhealthy	Sexual precocity	Collected by epidemiological investigation	1
PRJNA675895	20190602S3S4	SRR13077189	lncRNA\HiseqX	*Macrobrachium rosenbergii*	2.13E+07	201906	Gaoyou City, Jiangsu Province	Unhealthy	Sexual precocity	Collected by epidemiological investigation	1
PRJNA860710	20190611006-010	SRR27709255	lncRNA\HiseqX	*Penaeus vannamei*	4.28E+07	201906	Dongfang City, Hainan Province	Unhealthy	Black gill	Collected by epidemiological investigation	5
PRJNA860710	20190711-bingxia	SRR27709254	lncRNA\HiseqX	*Penaeus vannamei*	4.55E+07	201907	Wenzhou City, Zhejiang Provinc	Unhealthy	Empty stomach; empty intestine	Collected by epidemiological investigation	5
PRJNA860710	20200514GPL	SRR27709253	lncRNA\Novaseq 6000	*Penaeus vannamei*	3.04E+07	202005	Wenzhou City, Zhejiang Provinc	Unhealthy	Empty stomach; empty intestine	Collected by epidemiological investigation	5
PRJNA860710	20200601GPL	SRR27709252	lncRNA\Novaseq 6000	*Penaeus vannamei*	2.26E+07	202006	Hainan Province	Unhealthy	Empty stomach; empty intestine	Collected by epidemiological investigation	5
PRJNA860710	20200611GPL	SRR27709251	lncRNA\Novaseq 6000	*Penaeus vannamei*	3.25E+07	202006	Fujian Province	Unhealthy	Empty stomach; empty intestine	Collected by epidemiological investigation	5
PRJNA860710	20200821028dixin	SRR27709250	lncRNA\Novaseq 6000	*Macrobrachium rosenbergii*	3.42E+07	202008	Gaoyou City, Jiangsu Province	Unhealthy	Soft shell; empty stomach; emp	Collected by epidemiological investigation	1
PRJNA860710	20200821-tie10	SRR27709248	lncRNA\Novaseq 6000	*Macrobrachium rosenbergii*	3.97E+07	202008	Gaoyou City, Jiangsu Province	Unhealthy	Sexual precocity	Collected by epidemiological investigation	1
PRJNA860710	20200821-ZH3	SRR27709247	lncRNA\Novaseq 6000	*Macrobrachium rosenbergii*	3.28E+07	202008	Gaoyou City, Jiangsu Province	Healthy	NA	Collected by epidemiological investigation	1
PRJNA860710	20201124001_10Hun	SRR27709246	lncRNA\Novaseq 6000	*Penaeus chinensis*	4.25E+07	202011	Qingdao City, Shandong Provinc	Healthy	NA	Collected by epidemiological investigation	10
PRJNA860710	20210523001-009	SRR27709245	lncRNA\Novaseq 6000	*Penaeus vannamei*	3.24E+07	202105	Qingdao City, Shandong Provinc	Healthy	NA	Collected by epidemiological investigation	9
PRJNA860710	20210626-13	SRR27709244	lncRNA\Novaseq 6000	*Macrobrachium rosenbergii*	3.93E+07	202106	Gaoyou City, Jiangsu Province	Healthy	NA	Collected by epidemiological investigation	1
PRJNA860710	20210626-5-2	SRR27709242	lncRNA\Novaseq 6000	*Macrobrachium rosenbergii*	2.97E+07	202106	Gaoyou City, Jiangsu Province	Healthy	NA	Collected by epidemiological investigation	1
PRJNA860710	20210626-5-42	SRR27709241	lncRNA\Novaseq 6000	*Macrobrachium rosenbergii*	3.46E+07	202106	Gaoyou City, Jiangsu Province	Healthy	NA	Collected by epidemiological investigation	1
PRJNA860710	20210626-5-505	SRR27709240	lncRNA\Novaseq 6000	*Macrobrachium rosenbergii*	3.46E+07	202106	Gaoyou City, Jiangsu Province	Healthy	NA	Collected by epidemiological investigation	1
PRJNA860710	20210626-8	SRR27709239	lncRNA\Novaseq 6000	*Macrobrachium rosenbergii*	3.44E+07	202106	Gaoyou City, Jiangsu Province	Healthy	NA	Collected by epidemiological investigation	1
PRJNA860710	20210626-9	SRR27709238	lncRNA\Novaseq 6000	*Macrobrachium rosenbergii*	2.79E+07	202106	Gaoyou City, Jiangsu Province	Healthy	NA	Collected by epidemiological investigation	1
PRJNA860710	20210626-D1	SRR27709236	lncRNA\Novaseq 6000	*Macrobrachium rosenbergii*	3.57E+07	202106	Gaoyou City, Jiangsu Province	Unhealthy	Soft shell; empty stomach; emp	Collected by epidemiological investigation	1
PRJNA860710	20210626-D2	SRR27709235	lncRNA\Novaseq 6000	*Macrobrachium rosenbergii*	3.40E+07	202106	Gaoyou City, Jiangsu Province	Unhealthy	Soft shell; empty stomach; emp	Collected by epidemiological investigation	1
PRJNA860710	20210626-D3	SRR27709234	lncRNA\Novaseq 6000	*Macrobrachium rosenbergii*	3.42E+07	202106	Gaoyou City, Jiangsu Province	Unhealthy	Soft shell; empty stomach; emp	Collected by epidemiological investigation	1
PRJNA860710	20210626-T1	SRR27709233	lncRNA\Novaseq 6000	*Macrobrachium rosenbergii*	3.55E+07	202106	Gaoyou City, Jiangsu Province	Unhealthy	Sexual precocity	Collected by epidemiological investigation	1
PRJNA860710	20210626-T2	SRR27709232	lncRNA\Novaseq 6000	*Macrobrachium rosenbergii*	3.44E+07	202106	Gaoyou City, Jiangsu Province	Unhealthy	Sexual precocity	Collected by epidemiological investigation	1
PRJNA860710	20211126002Hun	SRR27709231	lncRNA\Novaseq 6000	*Penaeus chinensis*	3.33E+07	202111	Qingdao City, Shandong Provinc	Healthy	NA	Collected by epidemiological investigation	6
PRJNA860710	20211226001Hun	SRR27709230	lncRNA\Novaseq 6000	*Penaeus monodon*	3.15E+07	202112	Dongfang City, Hainan Province	Healthy	NA	Collected by epidemiological investigation	15
PRJNA860710	20211227001-004	SRR27709229	lncRNA\Novaseq 6000	*Exopalaemon carinicauda*	3.79E+07	202012	Qingdao City, Shandong Provinc	Healthy	NA	Collected by epidemiological investigation	4
PRJNA675895	A	SRR13077182	lncRNA\Novaseq 6000	*Macrobrachium rosenbergii*	1.29E+07	201806	Gaoyou City, Jiangsu Province	Unhealthy	Sexual precocity	Collected by epidemiological investigation; virus purified solution of tissue m	NA
PRJNA860710	C1	SRR27709228	lncRNA\Novaseq 6000	*Macrobrachium rosenbergii*	4.28E+07	201806	Gaoyou City, Jiangsu Province	Healthy	NA	Collected by epidemiological investigation	1
PRJNA860710	C2	SRR27709256	lncRNA\Novaseq 6000	*Macrobrachium rosenbergii*	5.93E+07	201806	Gaoyou City, Jiangsu Province	Healthy	NA	Collected by epidemiological investigation	1
PRJNA860710	C3	SRR27709226	lncRNA\Novaseq 6000	*Macrobrachium rosenbergii*	6.11E+07	201806	Gaoyou City, Jiangsu Province	Healthy	NA	Collected by epidemiological investigation	1
PRJNA860710	DX1-xunjiang	SRR27709286	lncRNA\Novaseq 6000	*Penaeus vannamei*	1.97E+07	201907	Wenzhou City, Zhejiang Provinc	Unhealthy	Empty stomach; empty intestine	Collected from infection experiment; virus homogenate of tissue mixture	5
PRJNA860710	DX2-3-xia	SRR27709225	lncRNA\Novaseq 6000	*Penaeus vannamei*	1.94E+07	201907	Wenzhou City, Zhejiang Provinc	Unhealthy	Empty stomach; empty intestine	Collected from infection experiment; virus purified solution of tissue mixture	5
PRJNA860710	DX3-3	SRR27709224	lncRNA\Novaseq 6000	*Penaeus vannamei*	1.88E+07	201907	Wenzhou City, Zhejiang Provinc	Unhealthy	Empty stomach; empty intestine	Collected from infection experiment; virus purified solution of tissue mixture	5
PRJNA860710	JP1	SRR27709259	lncRNA\Novaseq 6000	*Penaeus vannamei*	2.70E+07	201907	Wenzhou City, Zhejiang Provinc	Unhealthy	Empty stomach; empty intestine	Collected from infection experiment	5
PRJNA860710	JP2	SRR27709223	lncRNA\Novaseq 6000	*Penaeus vannamei*	3.27E+07	201907	Wenzhou City, Zhejiang Provinc	Unhealthy	Empty stomach; empty intestine	Collected from infection experiment	5
PRJNA860710	JP3	SRR27709222	lncRNA\Novaseq 6000	*Penaeus vannamei*	2.87E+07	201907	Wenzhou City, Zhejiang Provinc	Unhealthy	Empty stomach; empty intestine	Collected from infection experiment	5
PRJNA860710	JPD	SRR27709243	lncRNA\Novaseq 6000	*Penaeus vannamei*	2.31E+07	201907	Wenzhou City, Zhejiang Provinc	Healthy	NA	Collected from infection experiment	5
PRJNA675895	Luoshixiaduizhao1200	SRR13077190	lncRNA\Novaseq 6000	*Macrobrachium rosenbergii*	4.20E+07	201806	Gaoyou City, Jiangsu Province	Healthy	NA	Collected by epidemiological investigation; virus purified solution of tissue m	NA
PRJNA860710	PF201611CD-2	SRR27709221	lncRNA\HiseqX	*Penaeus chinensis*	4.10E+07	201806	Weifang City, Shandong Provinc	Unhealthy	Empty stomach; empty intestine	Collected by epidemiological investigation; virus purified solution of tissue m	1
PRJNA860710	s16	SRR27709219	lncRNA\HiseqX	*Trachypenaeus curvirostris*	5.16E+07	201811	Yellow Sea	NA	NA	Collected by epidemiological investigation	1
PRJNA860710	s20160707	SRR27709216	lncRNA\HiseqX	*Penaeus japonicus*	5.10E+07	201607	Binzhou City, Shandong Provinc	NA	NA	Collected by epidemiological investigation	1
PRJNA860710	s201904001	SRR27709215	lncRNA\HiseqX	*Penaeus vannamei*	4.43E+07	201904	Wenzhou City, Zhejiang Provinc	Unhealthy	Empty stomach; empty intestine	Collected by epidemiological investigation	5
PRJNA860710	s20200424	SRR27709214	lncRNA\Novaseq 6000	*Penaeus vannamei*	4.20E+07	202004	Zhangzhou City, Fujian Province	Unhealthy	Empty stomach; empty intestine	Collected by epidemiological investigation	5
PRJNA860710	s202005001	SRR27709213	lncRNA\Novaseq 6000	*Penaeus vannamei*	3.72E+07	202005	Ninghai City, Zhejiang Province	Unhealthy	Empty stomach; empty intestine	Collected by epidemiological investigation	5
PRJNA860710	s20200507001	SRR27709212	lncRNA\Novaseq 6000	*Penaeus vannamei*	3.54E+07	202005	Binzhou City, Shandong Provinc	Healthy	NA	Collected by epidemiological investigation	5
PRJNA860710	s20200507003	SRR27709211	lncRNA\Novaseq 6000	*Penaeus vannamei*	4.17E+07	202005	Binzhou City, Shandong Provinc	Unhealthy	Empty stomach; empty intestine	Collected by epidemiological investigation	5
PRJNA860710	s20200507006	SRR27709210	lncRNA\Novaseq 6000	*Penaeus vannamei*	4.25E+07	202005	Binzhou City, Shandong Provinc	Healthy	NA	Collected by epidemiological investigation	5
PRJNA860710	s20200815011	SRR27709270	lncRNA\Novaseq 6000	*Penaeus vannamei*	4.00E+07	202008	Rudong City, Jiangsu Province	Healthy	NA	Collected by epidemiological investigation	5
PRJNA860710	s26	SRR27709269	lncRNA\HiseqX	*Leptochela gracilis*	7.35E+07	201811	Yellow Sea	NA	NA	Collected by epidemiological investigation	1
PRJNA860710	S31	SRR27709220	lncRNA\HiseqX	*Metapenaeopsis dalei*	4.05E+07	201811	Yellow Sea	NA	NA	Collected by epidemiological investigation	1
PRJNA860710	T1	SRR27709267	lncRNA\Novaseq 6000	*Macrobrachium rosenbergii*	5.87E+07	201806	Gaoyou City, Jiangsu Province	Unhealthy	Sexual precocity	Collected by epidemiological investigation	1
PRJNA860710	T2	SRR27709217	lncRNA\Novaseq 6000	*Macrobrachium rosenbergii*	6.08E+07	201806	Gaoyou City, Jiangsu Province	Unhealthy	Sexual precocity	Collected by epidemiological investigation	1
PRJNA860710	T3	SRR27709266	lncRNA\Novaseq 6000	*Macrobrachium rosenbergii*	5.28E+07	201806	Gaoyou City, Jiangsu Province	Unhealthy	Sexual precocity	Collected by epidemiological investigation	1
PRJNA860710	T4	SRR27709265	lncRNA\Novaseq 6000	*Macrobrachium rosenbergii*	6.09E+07	201806	Gaoyou City, Jiangsu Province	Unhealthy	Sexual precocity	Collected by epidemiological investigation	1
PRJNA860710	TW	SRR27709264	lncRNA\Novaseq 6000	*Penaeus vannamei*	2.92E+07	201907	Wenzhou City, Zhejiang Provinc	Unhealthy	Empty stomach; empty intestine	Collected from infection experiment; virus purified solution of tissue mixture	5
PRJNA860710	TWD	SRR27709263	lncRNA\Novaseq 6000	*Penaeus vannamei*	2.87E+07	201907	Wenzhou City, Zhejiang Provinc	Healthy	NA	Collected from infection experiment; virus purified solution of tissue mixture	5

NA means not applicable
