# Supplementary material for: Enormous diversity of RNA viruses in economic crustaceans
Source: mSystems. 2024 Sep 27;9(10):e01016-24. doi: 10.1128/msystems.01016-24 (PMC11494968; doi:10.1128/msystems.01016-24)
Supplement: Table S3 — Primers used in this study. [file msystems.01016-24-s0004.pdf]

Supplementary Table 3. Primers used in this study.

No.	Virus name	Forward primer	Sequence 5'-3'	Return primer	Sequence 5'-3'	Product size
1	Beihai levi-like virus 30	levi-like virus F1	GAGGAAGCGCTTGGTAGTGT	levi-like virus R1	CTGCCAATCAAGGCCCCATA	558
2	Beijing sediment noda-like virus 3 segment 1	noda-like virus F1	GGTGTTGTCGGCTGGTCTAA	noda-like virus R1	GTGTTGCTCGTGTTTGGTCC	407
3	Beijing sediment noda-like virus 3 segment 2	noda-like virus F2	TGTCGTCTGTTGCGGATGAA	noda-like virus R2	TGAAGCCAGATAAGCGCACA	504
4	Penaeus vannamei noda-like virus 2 segment 1	noda-like virus F3	AACAATGTGATCCCCAGCGA	noda-like virus R3	ACGTTGCGCTGTAACCATTG	464
5	Penaeus vannamei partiti-like virus 2 segement 1	partiti-like virus F1	GCCCGGATCTAAATCCCCAG	partiti-like virus R1	GCCCGGATCTAAATCCCCAG	589
6	Penaeus vannamei partiti-like virus 2 segement 2	partiti-like virus F2	AACCACCCAGACTTAGTGCG	partiti-like virus R2	GAAGACGGCCTTCCTATCCG	453
7	Penaeus vannamei partiti-like virus 2 segement 3	partiti-like virus F3	CCACTGCTAACCCACCAGTT	partiti-like virus R3	AGCTATCTCTTGCGTGGCTC	428
8	Penaeus vannamei partiti-like virus 2 segement 4	partiti-like virus F4	CCACCAGTCACATCGTGGAA	partiti-like virus R4	CTACGCGCTCCAGAATGCTA	557
9	Covert mortality nodavirus segment 1	noda-like virus F5	GTTCAACGTCCTTTGTGCCC	noda-like virus R4	TTAGGCGCGCGATCATTTTG	501
10	Covert mortality nodavirus segment 2	noda-like virus F5	CCCCGGTACAAAATACCCCC	noda-like virus R5	GCCCTCGGAGTTAGGGTAGA	428
11	Crangon affinis narna-like virus 1	narna-like virus F1	CCGTAAAGGCCGTCTCCAAT	narna-like virus R1	GTGTAGTTTTCCCCTCGGCA	401
12	Crangon crangon flavivirus	flavivirus F1	CAAGCGTGGACCTTCAGTCA	flavivirus R1	CGAGTTCCTTCACCCGAACT	548
13	Crustacea hepe-like virus 1	hepe-like virus F1	ACGAACGAACGCAGGCTACAT	hepe-like virus R1	ACCGTCGCAATGCTCACAAGT	496
14	Eriocheir sinensis virus 1	Eriocheir sinensis virus F1	AGCAACAGCGAGAGAACGAA	Eriocheir sinensis virus R1	GTAGCGCAAGCGGTATTGTG	484
15	Euphausia pacifica bunya-like virus 1	bunya-like virus F1	TGTCAACAACATTGCCAGCG	bunya-like virus R1	AATGCACTGCTCTGACACGA	430
16	Euphausia pacifica noda-like virus 1 segment 1	noda-like virus F6	ACTGATCGGATCCCCTGGAA	noda-like virus R6	GGATAGCAGGGTCGATGGTG	527
17	Euphausia pacifica noda-like virus 1 segment 2	noda-like virus F7	TGGGAGGGTCACGGTAGAAT	noda-like virus R7	GGTGGTGGTAATTTGGCTGC	558
18	Exopalaemon carinicauda bunya-like virus 1	bunya-like virus F2	AGATGAAACCAAGTGGGGTCC	bunya-like virus R2	TGCCCTTTTGCATCTGACCA	589
19	Infectious precocity virus	flavivirus F2	ATGGAGGGGATCTTCGACCA	flavivirus R2	TTCCTCGTCAGTTGCTCCAC	448
20	Leptochela gracilis chuvirus-like virus 1	chuvirus-like virus F1	AGGGGGAACCTCTATCCACC	chuvirus-like virus R1	CGGACTAATCGGGACTCGTG	600
21	Macrobrachium rosenbergii bunya-like virus 1	bunya-like virus F3	AGGAGAGATGACAGTAGGGCT	bunya-like virus R3	CCTTCAACATTGCAGCACCC	590
22	Macrobrachium rosenbergii dicistro-like virus 1	dicistro-like virus F1	AGGGAACCCTCCTAGCGATT	dicistro-like virus R1	AAAATGGTCGGCATTGGTGC	522
23	Macrobrachium rosenbergii golda virus	ronivirus F1	GGCTCTTTATGTGCGCGAAG	ronivirus R1	GGTCACCGGGTACACCATTT	458
24	Macrobrachium rosenbergii hepe-like virus 1	hepe-like virus F2	CTACGCGATGAGCAAAACCG	hepe-like virus R2	CGTTTGGGCGAATCATCGTC	483
25	Macrobrachium rosenbergii hepe-like virus 2	hepe-like virus F3	TTCCGCTGCTTTGGATGCTA	hepe-like virus R3	TCCGTTGTCACAGTACGAGC	579
26	Macrobrachium rosenbergii hepe-like virus 3	hepe-like virus F4	TTACATTCCCAACGCGACCA	hepe-like virus R4	TCTCAACGCCGCTTGTAACT	576
27	Macrobrachium rosenbergii hepe-like virus 4	hepe-like virus F5	GTCCTATGGTTGCAGTGCCT	hepe-like virus R5	CATCAACGCGTTTTTCCCGT	412
28	Macrobrachium rosenbergii levi-like virus 1	levi-like virus F2	TCAATCCCCGCGATACTTCG	levi-like virus R2	CCTTGGAGCCGACATCTGTT	532
29	Macrobrachium rosenbergii marna-like virus 1	marna-like virus F1	ACACTCGAAGCGTTTTGTGC	marna-like virus R1	TGACAAACAACTGCCCACCT	404
30	Macrobrachium rosenbergii marna-like virus 2	marna-like virus F2	CACGTGACTCAAGTCGGTGA	marna-like virus R2	TCTGACAGCACCACGAGTTC	554
31	Macrobrachium rosenbergii narna-like virus 1	narna-like virus F2	AAGAACGCTATTGGTGCGGA	narna-like virus R2	CTCGCCATCTGGAAGATGCT	427
32	Macrobrachium rosenbergii narna-like virus 2	narna-like virus F3	CTACCATCCTTCGGAGCGTC	narna-like virus R3	GATCTGTGGCGGACTCGTAG	499
33	Macrobrachium rosenbergii noda-like virus 1	noda-like virus F8	CGAATTCCGTTGCTAAGGCG	noda-like virus R8	CGACATAGCTGTAGGGGCTG	413
34	Macrobrachium rosenbergii permutotetra-like virus 1	permutotetra-like virus F1	TTAAGCCGCCGACAAGAGTT	permutotetra-like virus R1	AGCTTACACACCGAGTCACG	444
35	Picornaviridae sp.	picorna-like virus F1	GTAGTTCCCGCCAAGACACA	picorna-like virus R1	AATGCACACCTCCACTAGCC	565
36	Macrobrachium rosenbergii picorna-like virus 10	picorna-like virus F2	CCAGAAGCTATGCAACGACG	picorna-like virus R2	TCGTCGCTAGGTTCAACACC	557
37	Macrobrachium rosenbergii picorna-like virus 11	picorna-like virus F3	CAACAAGCAGCCGCAGATTT	picorna-like virus R3	AGGCGCATAAGGTCGATCAG	489
38	Macrobrachium rosenbergii picorna-like virus 1	picorna-like virus F4	ACTCAGTACGACTCCGGTGA	picorna-like virus R4	CCACAGCTTCAGTGGGCATA	598
39	Macrobrachium rosenbergii picorna-like virus 2	picorna-like virus F5	TTTTGACGAGGCGGCTTTTG	picorna-like virus R5	CCGTGAGTCGTCGCGTATAA	600
40	Macrobrachium rosenbergii picorna-like virus 3	picorna-like virus F6	GATCTGACCCAGCGGTGAAT	picorna-like virus R6	CGGTATAAGAACGCTCGCCT	400
41	Macrobrachium rosenbergii picorna-like virus 4	picorna-like virus F7	TGGTCTCGTGGAGCAACTTC	picorna-like virus R7	TACGCTCTTCTCCAGTCGGA	403
42	Macrobrachium rosenbergii picorna-like virus 5	picorna-like virus F8	TAAAAGGCGATTGCGGGACT	picorna-like virus R8	AACCTGGCGAGCATTCTTGA	440
43	Macrobrachium rosenbergii picorna-like virus 6	picorna-like virus F9	GGTGCCATCGTTGGTGACTA	picorna-like virus R9	TGCGTTAGCAGGTGGATTGT	553
44	Macrobrachium rosenbergii picorna-like virus 7	picorna-like virus F10	TGCCGCAAACTGATCAAACG	picorna-like virus R10	TCGCTGTTGCGTTTCAAGTG	445
45	Macrobrachium rosenbergii picorna-like virus 8	picorna-like virus F11	GGTTTCGTCGGTTTGTGTGG	picorna-like virus R11	GCCGCTCTCTCACTCAACTT	410
46	Macrobrachium rosenbergii picorna-like virus 9	picorna-like virus F12	TGCTCAGACCGGAGGTGATA	picorna-like virus R12	TACCCATTCCGCACATGGTC	440
47	Macrobrachium rosenbergii solemo-like virus 1	solemo-like virus F1	GAACGAAGCCTCCACTGTGA	solemo-like virus R1	GTCGCAGCTAATTGCAGCTC	417
48	Macrobrachium rosenbergii tombus-like virus 1	tombus-like virus F1	AGACCGACGAAACAAGGTCC	tombus-like virus R1	TGAACAGCTCCCCGAGGATA	540
49	Macrobrachium rosenbergii tombus-like virus 2	tombus-like virus F2	GCCAGTTTCACACGCAACAT	tombus-like virus R2	GGGTTACATGAGCGTCCCAA	413
50	Macrobrachium rosenbergii tombus-like virus 3	tombus-like virus F3	CATCCTTGAGCGTGGTGACT	tombus-like virus R3	GTTGATGCCAAACGCCTTGT	451
51	Macrobrachium rosenbergii virus 1	Macrobrachium rosenbergii virus F1	AAAGTGAGCGAACGGGCTTA	Macrobrachium rosenbergii virus R1	TCATGCTCGCAACTGGTGAT	565
52	Macrobrachium rosenbergii virus 15	picorna-like virus F25	GACTTTGGCACGTTGCTCTG	picorna-like virus R25	ACCACCATCTTTTCCGGGAC	537
53	Penaeus chinensis levi-like virus 1	levi-like virus F3	CATGCGCGCACTTCTAACTC	levi-like virus R3	CCAGGCAAGCGAAAAGCAAT	472
54	Penaeus japonicus marna-like virus 1	marna-like virus F10	TCTCGAGCCACAAAGTGGAC	marna-like virus R10	TTCTCTAACCAATGCCGCGT	457
55	Penaeus japonicus marna-like virus 2	marna-like virus F3	ATTGGACAATCGTGGAGCGT	marna-like virus R3	TGGTGCTATGCGTGAATGGT	506
56	Penaeus japonicus marna-like virus 3	marna-like virus F4	AAACCATTAGGGCCGGACAG	marna-like virus R4	GGGCTCTCCCCCAGTAAAAC	403
57	Penaeus japonicus partiti-like virus 1 segment 1	partiti-like virus F5	TTCCAGGAAGCCCTTATGCG	partiti-like virus R5	CAATACGCTCTGCGGGATCT	556
58	Penaeus japonicus partiti-like virus 1 segment 2	partiti-like virus F6	ACGGAGTATCACCGGCAATG	partiti-like virus R6	TCCACCACCTTCGCAAACTT	565
59	Penaeus japonicus picorna-like virus 1	picorna-like virus F13	CACCGGCAGTCAAGTTGTTG	picorna-like virus R13	AACTCTCCCTTGCATGCTCC	515
60	Penaeus japonicus picorna-like virus 2	picorna-like virus F14	GGTACCACGGGGAAACAGAG	picorna-like virus R14	CAGCATGCGGTTTGATCGAG	414
61	Penaeus monodon picorna-like virus 1	picorna-like virus F15	CGCTCTGACTTTATGCGTGC	picorna-like virus R15	AAGTCACGTGGCGACATCAT	563
62	Penaeus vannamei reo-like virus 1	reo-like virus F1	AGCTGACAACACGACCTCTG	reo-like virus R1	AACGGCATCGCGGAGATATT	565
63	Penaeus vannamei chuvirus-like virus 1	chuvirus-like virus F2	CAAGTGAGCACTGGGCTGTA	chuvirus-like virus R2	GGTTCCTCGATCGGTGTGTT	556
64	Penaeus vannamei chuvirus-like virus 2	chuvirus-like virus F3	TGCGCTCAGCATAGTGAGTT	chuvirus-like virus R3	ACATGGCGGCCAACATTTTC	480
65	Penaeus vannamei levi-like virus 1	levi-like virus F4	AGAAGTACCGCTACACCCCT	levi-like virus R4	TCCTGCAGGTCTGAAACGTC	424
66	Penaeus vannamei levi-like virus 2	levi-like virus F5	GAAGTTCACGGGCAAACCAC	levi-like virus R5	GATGCGTCTGTCCTCAACCA	592
67	Penaeus vannamei marna-like virus 1	marna-like virus F5	AGTGTACTGTCCGCTTGTGG	marna-like virus R5	ACAGGTTGAATGTAGGGCCG	510
68	Penaeus vannamei marna-like virus 2	marna-like virus F6	CTGTGTCTAAGGCAACGGGT	marna-like virus R6	TACCGAGGGGGTCATACGTT	564
69	Penaeus vannamei marna-like virus 3	marna-like virus F7	ACGAAAACACGTCTGCGTTG	marna-like virus R7	GATGCCACACCGAACAGAGA	421
70	Penaeus vannamei marna-like virus 4	marna-like virus F8	GTTCTCGGCGCATTGTTTGT	marna-like virus R8	CCATAGCGTGTTCATGCAGC	559
71	Penaeus vannamei marna-like virus 5	marna-like virus F9	CTGGCTGGGGATGACCAAAT	marna-like virus R9	GTGGCAGATGACGATAGGGG	416
72	Penaeus vannamei narna-like virus 1	narna-like virus F4	AACGAGCGAAGGCTACAGTC	narna-like virus R4	AGTTGCCATCTGGACTTCCG	590
73	Penaeus vannamei narna-like virus 2	narna-like virus F5	CTCAGAAAGTTGTGCGCTGT	narna-like virus R5	GTCTCTCCGTAGAACGAGGG	488
74	Penaeus vannamei narna-like virus 3	narna-like virus F6	TTCGTTCCCAAGTCGTAGCC	narna-like virus R6	CCGCGGAATAGTCTCCTGAC	435
75	Penaeus vannamei narna-like virus 4	narna-like virus F7	CCAGCTGGTTTGGCTATGGA	narna-like virus R7	GTGAAAACCTGGACGTTGCC	565
76	Penaeus vannamei noda-like virus 1	noda-like virus F9	CCATCTTGAAGCGCGATGTG	noda-like virus R9	GGGTCAAACGCAAAAGGTCC	400
77	Penaeus vannamei partiti-like virus 1 segment 1	partiti-like virus F7	AGCGATCTATCAGGCACACG	partiti-like virus R7	TCTGTCGTTCCTAAAGGGCG	423
78	Penaeus vannamei partiti-like virus 1 segment 2	partiti-like virus F8	AGAATCCCGCATCATTCGCT	partiti-like virus R8	CCATTGGGGAGAGGTGGTTC	556
79	Penaeus vannamei picorna-like virus 1	picorna-like virus F16	TAGAGTGTGCGTCGGCAAAT	picorna-like virus R16	ATGCTCGTGGAACTTACGCA	586
80	Penaeus vannamei picorna-like virus 2	picorna-like virus F17	AGTGGCCGTTCCTGGTATTG	picorna-like virus R17	GTCATACCGCAATGAACCGC	457
81	Penaeus vannamei picorna-like virus 3	picorna-like virus F18	TACTGTGTTGGCCGATGCTT	picorna-like virus R18	CGAAAGCACAAACGGCATCA	521
82	Penaeus vannamei picorna-like virus 4	picorna-like virus F19	AGGTGCGGAATTGGGTTAGG	picorna-like virus R19	GCCAGCGCTATAAAAGCCAC	584
83	Penaeus vannamei picorna-like virus 5	picorna-like virus F20	CATTTGGCGAAAGGGCAACA	picorna-like virus R20	ATGCGGGTCATGAATGGTGT	561
84	Penaeus vannamei picorna-like virus 6	picorna-like virus F21	TACTCAGTACGCCCGAGACA	picorna-like virus R21	TACTCAGTACGCCCGAGACA	493
85	Penaeus vannamei qinvirus-like virus 1	qinvirus-like virus F1	AATGGGCCGAGTGATGTGAG	qinvirus-like virus R1	GATGCACCACCTGGCTTAGT	506
86	Penaeus vannamei tombus-like virus 1	tombus-like virus F4	TCTGAGGCCCCGAGAACATA	tombus-like virus R4	ATAACGTGACGGTTGGTGCT	597
87	Penaeus vannamei tombus-like virus 2	tombus-like virus F5	GTGTCTTTTCGCGCAAGGTT	tombus-like virus R5	TCCCCTGTAGGTTCCGTAGG	540
88	Portunus trituberculatus chuvirus-like virus 1 sgement 1	chuvirus-like virus F4	ACTCCAATGGCATCAGGTGG	chuvirus-like virus R4	CGAGAGCAGTAGCATACGCA	481
89	Portunus trituberculatus chuvirus-like virus 1 sgement 2	chuvirus-like virus F5	CCCCAGGGCATCTGAACATT	chuvirus-like virus R5	GGTTGAAACGCAAGTTCCCC	485
90	Portunus trituberculatus picorna-like virus 1	picorna-like virus F22	TGGGATCGTCAAGTGCCTTC	picorna-like virus R22	ACAGTAAGACCGCACTCAGC	512
91	Portunus trituberculatus reo-like virus 1	reo-like virus F2	TTTCCCGTTGGCACAGTTCT	reo-like virus R2	GTATGTTGGAAACGCTCCGC	427
92	Portunus trituberculatus solemo-like virus 1	solemo-like virus F2	AGTTTGTACGCTGTCTGCGA	solemo-like virus R2	TGAGCGGCCGTTATGAACTT	560
93	Portunus trituberculatus tombus-like virus 1	tombus-like virus F6	ACGCAGGAAAACCAAATGGC	tombus-like virus R6	GATCCAGCGTCTCTCGTCTG	406
94	Shahe isopoda virus 5	Macrobrachium rosenbergii virus F2	GAGTTCAGCACTGTTCGGGA	Macrobrachium rosenbergii virus R2	CGGTGTTTCCCTCCACGTAA	410
95	Trachypenaeus curvirostris picorna-like virus 1	picorna-like virus F23	CTGGCAACAGTTGGCTTGAC	picorna-like virus R23	CTAGTCGCACTACCTGAGCG	437
96	Oriental wenrivirus 1 segement L	bunya-like virus F4	AATACGTGTGTTCCGGCCAT	bunya-like virus R4	TTCGAGGAAGCTTTCAGCGT	436
97	Oriental wenrivirus 1 segement M	bunya-like virus F5	GCTCATAGTACCTCCACCGC	bunya-like virus R5	TTTCGGCCCGTCGTAATCAA	560
98	Oriental wenrivirus 1 segement S1	bunya-like virus F6	GGTGGCCATTCTGTGTGAGA	bunya-like virus R6	TGGGATGTGGGTCGAGTTTG	439
99	Oriental wenrivirus 1 segement S2	bunya-like virus F7	ACACCTGAATTCGGCATGGT	bunya-like virus R7	TGCTCCCACCACTGATTGAC	467
100	Wenzhou channeled applesnail virus 2	picorna-like virus F26	GTGGAGCTTTCTGCTCTGGT	picorna-like virus R26	TCGATTGCATACTCGCGACA	418
101	Wenzhou picorna-like virus 41	picorna-like virus F24	AGCCCGATACGCCTTAACTG	picorna-like virus R24	CGCCTGTGAAATCCCCTGAT	480
102	Wenzhou shrimp virus 8	Penaeus vannamei virus 1	GAATCTGCGCGAGCTCTTTG	Penaeus vannamei virus 1	GGGTTGAAAAACTCCGTGGC	590
